# Cryptic Vocal Communication in the Proboscis Bat (*Rhynchonycteris naso*)

**DOI:** 10.1111/nyas.70345

**Published:** 2026-07-21

**Authors:** Lena E. Dressler, Karl‐Heinz Frommolt, Mirjam Knörnschild, Martina Nagy

**Affiliations:** ^1^ Museum Für Naturkunde, Leibniz Institute For Evolution and Biodiversity Science Berlin Germany; ^2^ Freie Universität Berlin, Institute for Biology Berlin Germany; ^3^ Humboldt‐Universität Zu Berlin, Institute for Biology Berlin Germany; ^4^ Smithsonian Tropical Research Institute Balboa Panama

**Keywords:** acoustic crypsis, bats, Chiroptera, selection pressure, signature, social vocalization, vocal communication

## Abstract

Cryptic‐living animals face the trade‐off between communicating effectively while avoiding detection. To examine how this trade‐off shapes vocal behavior, we studied the vocal repertoire of the cryptic‐living, neotropical proboscis bat (*Rhynchonycteris naso*; Emballonuridae). *R. naso* produces 22 syllable types and 17 vocalization types, including song. One common social vocalization type resembles modified echolocation calls and may also provide sensory information. Vocalizations shaped by natural selection appeared adapted to minimize predation risk: they are short, span broad frequency ranges, have limited detection ranges, and are mostly emitted during rocking, a behavior that maintains crypsis. In contrast, sexually selected vocalizations such as song are more conspicuous, highlighting a tension between effective signaling and remaining undetected. A phylogenetic analysis across 38 emballonurid bat species revealed that *R. naso* emits exceptionally high‐frequency echolocation calls, beyond expectation from body size alone, and beyond the hearing range of most predators and prey, probably reflecting acoustic crypsis. The high‐frequency echolocation calls of *R. naso* reduce detection distances for hearing moths and likely increase capture success. Overall, our study shows that *R. naso* employs diverse acoustic adaptations to communicate effectively while minimizing detection risk, offering rare insight into the evolution of vocal behaviors in cryptic species.

## Graphical abstract

Proboscis bats (*Rhynchonycteris naso*) balance communication and crypsis through distinct vocal strategies. Naturally selected calls are high‐frequency, short‐range, and produced during motion, reducing predator detection. In contrast, sexually selected signals are more conspicuous. Echolocation calls exceed allometric expectations and are poorly detected by prey, supporting acoustic crypsis. Together, these adaptations allow *R. naso* to communicate effectively while minimizing detection risk.

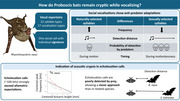

## 1 Introduction

Animals face the trade‐off between successfully communicating with conspecifics while remaining undetected by predators. This trade‐off becomes particularly acute during parental care or the mating season. Crypsis can be an effective antipredator adaptation for animals living in environments with permanent or unpredictable predation risk [1]. It refers to camouflage, which makes organisms harder to detect and localize [1, 2]. While visual camouflage is the most widely known form, crypsis can also act through other sensory modalities, such as sound, odor, or electroreception [1]. Beyond vision, most evidence has been gathered for acoustic crypsis. This phenomenon has been described in various taxa, including birds, insects, and mammals, especially cetaceans [1, 2].

Acoustic crypsis can be achieved through various mechanisms, such as using higher frequencies that attenuate quickly and reduce detection distance [3, 4, 5, 6, 7] or by lowering call amplitude to shorten the signaling range [8, 9, 10]. Some animals produce exceptionally quiet courtship signals to enable private communication [11, 12]. Others reduce vocal activity in the presence of predators [13, 14, 15, 16], or synchronize calls to dilute predation risk [17]. Habitat choice can further support acoustic crypsis, either by avoiding predators or by exploiting environments with poor sound propagation [18, 19].

Certain insects preyed upon by echolocating bats exhibit unique adaptations to reduce their acoustic detectability [20], such as flying close to cluttered vegetation to blend into background echoes [21], resting on sound‐absorbing surfaces to reduce acoustic shadow effects [22], or having sound‐absorbing scales that reduce their acoustic signature [23]. Although echolocation signals and their role in crypsis have been studied on the prey side (eared moths), acoustic crypsis has received little attention on the bats’ side. Only a few examples of acoustic crypsis in bats are currently known. The barbastelle bat (*Barbastella barbastellus*) uses low‐amplitude echolocation calls that are belatedly detected by eared moths, resulting in higher capture rates [24]. Low‐intensity and high‐frequency calls (higher than moths can hear) have been found in other bat species as well [25].

Given bats’ dependency on acoustic signals for both hunting and communication and their exposure to diverse predators, bats represent an ideal taxon [26] to explore the potential for acoustic crypsis and its role in shaping vocal behavior from the perspective of both predators and prey. To date, evidence for crypsis in bats has been largely limited to the visual domain. Some cryptic bats have background‐matching body coloration, resembling leaves or the bark of a tree. Other cryptic bats have disruptive markings, which makes it more difficult to detect the contour of the bat [27, 28, 29, 30, 31]. However, acoustic crypsis has been poorly described in bats, and the vocal repertoires of only a small fraction of bat species have been documented [32, 33, 34, 35], none of which belong to a cryptic bat species.

To expand current understanding, we studied the vocal communication of the cryptic bat species *Rhynchonycteris naso* (Wied‐Neuwied 1820), also known as the proboscis bat. This emballonurid, neotropical bat species roosts in well‐lit places, such as the outside of trees or on artificial structures [36]. Thus, the predation risk might be high during the day as roosts are easily accessible to a wide range of predators such as birds, small mammals, and snakes. To cope with this, proboscis bats have a cryptic lifestyle, affecting their appearance and behavior. They have conspicuous hair tufts on their forearms and two light‐colored, wavy lines on their back, which could help to disrupt their contour. The fur is multicolored and looks similar to the bark of popular roosting trees [37]. Furthermore, *R. naso* seldom moves in their day roost, which could help to maintain the visual camouflage. Sometimes, when they need to move to urinate, defecate, stretch, or groom, they do so in synchrony and during a wind gust to resemble lichen moving in the wind. This behavior is called *rocking* [38].

Roost choice and crypsis shape key aspects of *R. naso*’s life, including social structure and reproduction. Similar to other closely related emballonurids [39], territorial male *R. naso* secure most copulations. However, they tolerate other males in their territory, likely to avoid conspicuous aggression, resulting in multimale multifemale social groups. These groups are highly stable, with individuals showing extremely high group fidelity, being present in 97%–100% of observations over multiple years [40]. Dominant males guard estrous females directly [40]. Harsh and variable conditions, such as extreme heat in their exposed day‐roosts, often force entire groups to relocate (e.g., to a nearby tree or the other side of a house), transferring dominance to a new male in the new territory [40, 41]. Thus, *R. naso*’s appearance, movement, and social dynamics are shaped by visual crypsis and thermoregulatory demands. Yet, despite its exposed roosts, the species is vocally active, especially during the mating season, when it emits vocalizations audible to humans. This raises the question of whether and how *R. naso*’s vocalizations balance the need for concealment with effective social communication. Beyond social communication, we also explored the potential for acoustic crypsis in the unusually high‐frequency echolocation of *R. naso* (~100 kHz). Remaining undetected while echolocating may reduce detection by predators and prey, thereby potentially increasing foraging success in proboscis bats.

To address this, we studied the vocal repertoire of *R. naso* and explored ecological and evolutionary factors potentially influencing its structure. We hypothesized that *R. naso* exhibits adaptations consistent with acoustic crypsis to avoid detection by predators. More precisely, we hypothesized that the vocal repertoire is dominated by high‐frequency syllables, which attenuate quickly and fall outside most predators’ hearing range, thus reducing detection risk and enabling inconspicuous communication. Additionally, we hypothesized that proboscis bats shift their vocal activity to rocking periods to reduce detectability by unintended receivers. We expected that sexually selected vocalizations deviate from this pattern, as selection should favor more conspicuous signals in this context. We also hypothesized that social vocalizations serve a dual function by providing information about individual identity, sex, and colony affiliation as well as sensory information about the environment. Finally, we hypothesized that the high‐frequency echolocation calls of *R. naso* allow the bats to remain cryptic during foraging, thus reducing the distance at which eared insect prey may detect an approaching bat.

## 2 Materials and Methods

### 2.1 Sound Recordings and Behavioral Observations

We observed and recorded free‐ranging proboscis bats in Guanacaste, Costa Rica (10°37’ N/85°28’ W), in five free living colonies during their mating season [40] in March—April of 2022 and 2023, and October—November of 2022 and 2023. These data were used to describe the species’ vocal repertoire. Additionally, sound recordings were also conducted at the La Selva field station of the Organization for Tropical Studies (Costa Rica, Province Heredia, 10°25’ N/84°00’ W). Here, we recorded bats in two night‐roosts in September 2010 as well as in June—August 2006 and 2007, and during the mating season in October 2015.

This study was approved by the legal authorities of Costa Rica (MINAET Ministerio del Ambiente, Energia y Telecomunicaciones, SINAC Sistema Nacional de Areas de Conservación and, Comisión Nacional para la Gestión de la Biodiversidad—CONAGEBIO. Permit numbers: 130–2010‐SINAC, R‐006–2015‐OT‐CONAGEBIO, N° R‐SINAC‐ACG‐PI‐015‐2022; R‐045‐2022‐ OT‐CONAGEBIO).

At all study sites, we banded bats with colored plastic rings (AC Hughes Ltd., UK and Avian ID, size XCS) on their forearms to individually identify them in their day‐ and night‐roosts with binoculars and on video recordings. Bats were captured for banding with mist nets (Ecotone monofilament, Gdynia, Poland) when emerging from or returning to their roosts at dusk or dawn. We set the mist nets several meters away from the roosts, to make sure that bats did not associate the capturing event with a potential threat to their roosting sites.

For describing the vocal repertoire, we recorded vocal communication and associated social behaviors in five free‐living colonies of proboscis bats during the day. Social group size ranged between 8 and 52 individuals (Table S1). Each social group had a balanced sex ratio. During the observation periods, multiple pups were present. The colonies were located on trees and artificial structures and habituated to the presence of human observers. We used a condenser ultrasound microphone (CM16/CMPA, frequency range 2–200 kHz) on a tripod that was connected to an ultrasound recorder (USG 116Hm mobile; both Avisoft Bioacoustic, Glienicke N., Germany) controlled by a tablet (Dell Venue 8, Windows) or a laptop (Lenovo S21e ‐20, Windows). The Avisoft‐RECORDER software (v4.2.05, Specht, Avisoft Bioacoustic) was used to start the recording manually (with pretrigger function), with a sampling frequency of 500 kHz and a resolution of 16 bits. In addition, we used two video cameras (JVC‐Camcorder GC‐PX100, Sony Handycam DCR‐SR190) that ran synchronously with the audio recording. In the case of bad light conditions, infrared lights were used during video recording (SONY HVL‐IRC or Sionyx 940 nm LED). In total, we had access to data from 258 recording sessions during the day and 24 recording sessions during the night. A recording session lasted approximately 2 h. We rotated the recording schedule across the time of day and colonies.

### 2.2 Extracting Vocalizations and Classification Process

We defined the following categories: Syllables are the smallest unit of a vocalization surrounded by silence. A monosyllabic vocalization consists of one syllable. A monosyllabic composite vocalization has one syllable, which is a fused combination of at least one noisy and one tonal component. A multisyllabic simple vocalization contains multiple syllables of one type (often called train or sequence), which are regularly spaced. A multisyllabic complex vocalization consists of multiple syllables belonging to at least two different syllable types. Syllables and vocalizations were grouped into types based on their visual appearance and social function. To delineate vocalization types, we used intersyllabic intervals: successive syllables were classified as belonging to the same vocalization type when within‐type intervals were at least twofold shorter than intervals separating two consecutive vocalization types.

We scanned the recordings visually using the software Avisoft SASLab Pro (5.3.00, R. Specht, Avisoft Bioacoustic). After this, we manually extracted vocalizations/syllables with a high signal‐to‐noise ratio and sorted them according to their visual appearance. We aimed for data from different days, seasons, and colonies to capture as much variation as possible. We selected 21−58 examples per syllable type, and 7−54 per vocalization type (Tables S2 and S3). Not all recorded vocalizations/syllables could be assigned to one of the defined vocalization/syllable types because they occurred rarely.

### 2.3 Acoustic Parameter Measurement

We measured the acoustic parameters of the extracted vocalizations and syllables in Avisoft SASLab Pro semi‐automatically. For this, we preprocessed each file with bandwidth filtering and, if needed, noise reduction. In some cases, vocalizations of other animals or echoes had to be removed manually from the spectrogram with the *eraser* function in Avisoft SASLab Pro prior to measuring acoustic parameters. This procedure leaves the remainder of the sound file unchanged and serves only to prevent unintended elements from being included in measurements. We determined the start and end of each syllable/vocalization based on the oscillogram. In the case of multisyllabic vocalizations, we measured individual syllables as well as the total vocalization. Whenever we measured tonal vocalizations, we selected the clearest and most emphasized harmonic for measurement and subsequently converted all measurements to the fundamental frequency (F0) for comparison. This ensured that all frequency‐related parameters are expressed on the same scale (F0), even when measured on different harmonics. Measurements of nontonal (noisy and composite) syllables or over the entire vocalization were conducted for all harmonics. The spectrogram was computed by a 1024‐point FFT with an overlap of 98.43% using a Hamming window. This resulted in a frequency resolution of 488 Hz and a temporal resolution of 0.032 ms. We measured six spectrum‐based parameters, namely, peak, minimum, and maximum frequency, bandwidth, entropy, and harmonic‐to‐noise‐ratio, at seven locations distributed equally over the entire syllable/vocalization (start, center, end, and four intermediate locations). Furthermore, we extracted the average of the spectrum‐based parameters over the entire syllable/vocalization (mean) and over the maximum amplitude of frequencies encountered during the entire syllable/vocalization (meanentire). We measured two temporal parameters, namely, duration and interval. For simple multisyllabic vocalizations (trains), all values were averaged per train. The measured values were exported into Microsoft Excel (Microsoft Corporation, 2018).

### 2.4 Statistical Analysis of the Vocal Repertoire

Tonal and nontonal syllables were analyzed separately due to their fundamentally different acoustic structure and the ability to reliably extract frequency‐based parameters and conversion to the fundamental frequency for only tonal syllables. We conducted two principal component analyses (PCAs) to reduce parameter intercorrelation and to derive frequency curvatures (PCA 1) and entropy curvatures (PCA 2) for subsequent discriminant function analysis (DFA). The first PCA included frequency‐related parameters describing frequency curvature. Three components (eigenvalues > 1) explained 89.3% (tonal) and 61.7% (nontonal) of the variance. The second PCA included entropy parameters and yielded one component with an eigenvalue >1, explaining 75% (tonal and nontonal) of the variance. Suitability for PCAs was confirmed in all cases (tonal: PCA 1: KMO = 0.878, Bartlett's χ^2^ = 91193.87, df = 378, *p* < 0.001; PCA 2: KMO = 0.765, Bartlett's χ^2^ = 3543.03, df = 21, *p* < 0.001; nontonal: PCA 1: KMO = 0.662, Bartlett's χ^2^ = 6659.59, df = 378, *p* < 0.001; PCA 2: KMO = 0.28, Bartlett's χ^2^ = 540.75, df = 21, *p* < 0.001). All PCAs used varimax rotation, and standardized component scores were computed using Bartlett's regression approach.

We used separate DFAs for tonal and nontonal syllables to test whether visually grouped syllable types (in total 22 types, 681 syllables) could be statistically differentiated. Group sizes were weighted by prior probabilities to account for unequal sample sizes, and classification accuracy was estimated via leave‐one‐out cross‐validation. Eleven original acoustic parameters (duration; distance to maximum amplitude; energy; minimum, peak, and maximum frequency averaged over the element; peak frequency at start, center, and end of the element; entropy and harmonic to noise ratio averaged over the element) and four principal components (the first three of PCA1 and the first of PCA2) were included in the DFA. By pooling structurally similar syllable types, we gained 12 classes of syllable types. For these classes, we conducted additional DFAs to test whether these broader, structurally similar groupings were also acoustically distinct. Settings and assumptions remained unchanged. Binomial tests were used to evaluate whether classification success exceeded chance level. Analyses were conducted in SPSS (v29.0.2.0, IBM SPSS Statistics).

### 2.5 Behavioral Context Assignment

During the recording sessions in Guanacaste, we obtained audio and video material and conducted live behavioral observations in the day roosts of the habituated bats. Because *R. naso* produces vocalizations with a conspicuously open mouth, vocal emissions could be assigned unambiguously to specific individuals. This strategy was used to assign the behavioral context of each vocalization type. Only unambiguously identifiable cases were included, and assignments were made manually without dedicated behavioral coding software. In general, we used the following five behavioral categories: rocking (simultaneous movement from side to side during the occurrence of a gust of wind, often accompanied by urinating, grooming, defecation, or stretching), mother–pup interaction, aggression, flight/alert, and mating. Aggressive calls and songs are predominantly produced in a sexually selected context. Songs occur almost exclusively during the mating seasons. Aggressive vocalizations typically occur during physical competition among males or when females reject male copulation attempts [40]. Vocalizations for mother–pup communication, during low‐arousal context (e.g., during periods of autogrooming and absent aggressive interactions), and echolocation calls were classified as predominantly naturally selected vocalizations. Contexts for syllable types were determined based on their most frequent occurrences in vocalization types with their respective predominant selection pressure.

To investigate whether vocalizations were produced more often during rocking events, we reviewed a subset of the vocalizations used for repertoire analysis (*n* = 202) alongside field notes and video recordings and determined whether they were emitted during group rocking events or during periods when the bats were motionless. Each rocking event was counted only once.

### 2.6 Personal Information in *Hooks*

To assess the strength of individual differences in acoustic parameters of the most frequently produced social vocalization type (*Hooks*), we conducted a DFA (six individuals, 17–47 *Hook* sequences per individual). *Hooks* were produced by individuals of both sexes across multiple contexts and provided the most suitable data set for testing whether social calls encode individual, sex, and colony information. The acoustic data were recorded in La Selva, Costa Rica, from two colonies in September 2010. Only individuals for which at least 17 *Hook* sequences had been recorded during multiple nights were used for acoustic measurements. Eleven acoustic parameters were selected for the DFA, namely, duration, interval, distance to maximum amplitude, peak frequency at the start and end, and averaged over the element, and frequency curvature PCs 1–5. Frequency curvature PCs 1–5 are derived parameters obtained by calculating a principal component analysis on 60 frequency parameters distributed equally over each *Hook* (peak, minimum, and maximum frequency as well as bandwidth calculated at 15 different locations spread over the entire *Hook*). All parameters were checked for multicollinearity and included simultaneously in the DFA. A subset validation procedure was used to estimate the correct classification success, with 50% of the data randomly assigned to the training and test data set, respectively. The DFA was adjusted to the unequal number of analyzed recordings per individual by computing group sizes based on prior probabilities. We subsequently performed a binomial test to check whether the obtained classification success was better than a random classification. To test for acoustic differences in *Hook* between colonies or sexes, we conducted MANOVAs with the above‐mentioned 11 dependent variables and sex or colony ID as fixed factors (colony 1: one male, three females; colony 2: one male, one female). Tests were conducted in SPSS (v.20; IBM SPSS Statistics, Chicago, IL, USA).

### 2.7 Body Size and Peak Frequency Phylogenetic Correlation

We used a phylogenetic comparative approach to test whether the echolocation frequency of *R. naso* aligns with allometric expectations based on body size for emballonurid bats. As a proxy for body size, we used forearm length, selecting the mean value recorded for each species. Data were taken from published sources (Table S4). We extracted a phylogenetic tree with node ages from a bat supertree with Bayesian dating [42]. The supertree was pruned to include emballonurid bats for which we had forearm sizes (*n* = 38) using the *ape* [43] and *Geiger* [44] packages in R version 4.3.1 (R Core Team 2023). Subsequently, a phylogenetic comparative approach based on Ornstein–Uhlenbeck models of trait evolution was applied [45]. Ornstein–Uhlenbeck models of trait evolution can model the rate of adaptation and evolution of a trait (e.g., signal frequency) toward an optimal state as a linear function of a predictor (e.g., body size); this makes them well‐suited to test if there was an adaptive correlated evolution between two traits, such as signal frequencies and body size. We calculated phylogenetically controlled regression slopes with signal frequency as trait and body size as predictor. Phylogenetic tests were conducted in R (R v3.6.0; R Core Team 2019) using the SLOUCH package (stochastic linear Ornstein–Uhlenbeck models for comparative hypotheses).

### 2.8 Acoustic Detection Range Calculations and Loudness Comparison of Social Calls

To estimate the detection distances of different echolocating bat species, we used the following sonar equation with source levels (SLs) of the echolocation calls (extracted from [46, 47]); target strength (TS) of 5, 20, and 40 dB referring to a big, medium, and small prey item (as described in [46]) and spherical and atmospheric transmission loss (TL) multiplying the distance by two (sound traveling and echo returning). We assumed an echo detection threshold of 20 dB [48] for bats. To resemble the ambient conditions of a tropical dry forest, we used a temperature of 28°C, humidity of 65%, and air pressure of 101,325 Pa in the calculations.

$$SL\;\left(echo\right)=SL\left(call\right)-TS-TL\left(atmos.\right)-TL\;\left(spherical\right)$$

Spherical TL for the bat detection distance (two‐way) is calculated with:

$$TL\;\;\left(spherical\right)=2*20*log10\left(distance\right)$$

Atmospheric TL for the bats’ detection distance (two‐way) is calculated for the dominant frequency of their echolocation calls with the following equation, where *a* is the pure‐tone sound attenuation coefficient for atmospheric absorption according to the international standard (ISO 9613‐1) [49].

$$TL\;\;\left(atmos.\right)=a\;*\;\left(2\;*\;distance\right)$$

Hearing moths are part of *R. naso's* diet. For example, members of the family Noctuidae can hear above 90 kHz [50, 51], enabling them to hear the echolocation calls of bats. To calculate at what distances hearing moths can detect bats, we extracted hearing thresholds of 11 neotropical nocturnal moth species (from [51]), set the TS to zero, and used only a one‐way distance for sound transmission. We calculated at which distances moths could detect three different bat species. The three species are closely related, live sympatrically, and include hearing moths as part of their diet: *R. naso* (99.6 kHz, 120 dB SPL at 0.1 m, 7.3 m/s flight speed), *Saccopteryx bilineata* (44.7 kHz, 124 dB SPL at 0.1 m, 7.3 m/s flight speed), *Saccopteryx leptura* (52.2 kHz, 122 dB SPL at 0.1 m, 7.3 m/s flight speed). Flight speed is only known from *S. bilineata*, similar speed was assumed for both other species [52]. SLs and peak frequencies were taken from [47].

To estimate the active space of *R. naso* vocalizations, that is, how far the vocalizations propagate, we used the same ambient settings. For this, we used one‐way distances for sound transmission, set the TS to zero, and assumed a detection threshold of 20 dB. We measured the peak frequency of the loudest harmonic for each tonal syllable type in the vocal repertoire of *R. naso*. When two different harmonics were often emphasized at a similar rate, the peak frequencies and the active space of both harmonics were measured separately. The peak frequency of all harmonics together was measured for nontonal syllable types. To estimate the sound pressure level of the individual calls, we used an indirect method. The starting point is the assumption that the sound pressure level of the *Hooks* is 120 dB (RMS relative to 20 µPa) at a distance of 0.1 m and, consequently, 100 dB at a distance of 1 m, according to [47]. We determined the magnitude of the deviation in amplitude of the individual vocalization types relative to the *Hooks* in the same recording. All measurements were conducted on original audio files recorded with identical gain and recording settings, without noise filtering or manual editing. First, a reference amplitude was obtained from echolocation‐like *Hook* calls. The three loudest, nonclipped, and nonoverlapping *Hooks* within a file were selected, and their peak‐to‐peak amplitudes were measured using automated parameter extraction in Avisoft SasLab Pro (version 5.3.2). The mean peak‐to‐peak value was calculated and converted to amplitude by dividing by two. Based on this value, a synthetic sinus tone was generated. A power spectrum was computed from this tone (Hamming window, FFT 1024), and the amplitude in dB of the dominant spectral peak was extracted. Subsequently, we selected the three loudest nonclipped and nonoverlapping syllables for each syllable type. For each syllable, a power spectrum with identical settings was computed, and the peak dB value was extracted. The mean value across the three syllables was calculated. The difference in dB between the syllable and the *Hook* reference was then used to obtain a rough estimate of SL (re. 20 µPa). Specifically, the absolute difference was subtracted from 100 dB to obtain the estimated SL for each syllable type. This procedure was repeated across 7–12 recordings per syllable type, and mean SLs were calculated. The estimated values were used as a proxy for loudness (Table S5). Our amplitude and range estimates should be regarded as approximations rather than as precise values. Our high‐quality microphone had a flat frequency response between 30 and 130 kHz, but two syllable types fell below 20 kHz, where the microphone's sensitivity is more variable. Therefore, the validity of the range estimation of these frequencies under 20 kHz is limited.

### 2.9 Vocal Activity during the Mating versus Nonmating Season

To show that *R. naso* is most vocal during the mating season, we compared the number of sound files containing at least one social call for a small but representative subset of recordings conducted during the mating season (October 2015) and outside of the mating season (June−August 2006 and 2007). Recordings were conducted in a large colony (2006: 23 bats, 2007: 31 bats, 2015: 34 bats) at La Selva Biological Station in Sarapiquí. We screened eight 1 h‐long recording sessions, equally distributed over the whole day, for social calls and compared both scenarios.

## 3 Results

### 3.1 The Vocal Repertoire of Proboscis Bats

**FIGURE 1:**
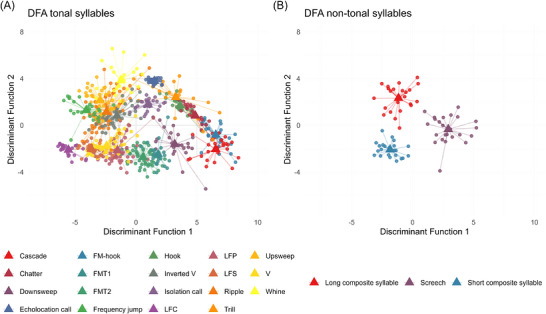
Discriminant function analysis of tonal and nontonal (noisy and composite) syllables. Triangles show centroids of syllable types. Datapoints are connected to the centroid for better visualization. Only the first two discriminant functions are shown. Abbreviation: DFA, discriminant function analysis.

The vocal repertoire of *R. naso* consisted of at least 22 distinct syllable types (Figure 1). Most syllable types were purely tonal, one was purely noisy, and two syllable types combined noisy and tonal parts. Because acoustic measurements relied on harmonic structure, and noisy or composite syllables lacked detectable harmonics, two separate statistical analyses were performed. A DFA for the purely tonal syllables confirmed our visual classification: 77.2% of syllables were correctly assigned to the respective types, significantly exceeding the chance level of 5.3% (binomial test, *p*<0.001). For the three nontonal syllables, the DFA yielded a classification success of 93.0%, also significantly above the chance level of 33.0% (binomial test, *p*<0.001, more details in Tables S6 and S9).

**FIGURE 2:**
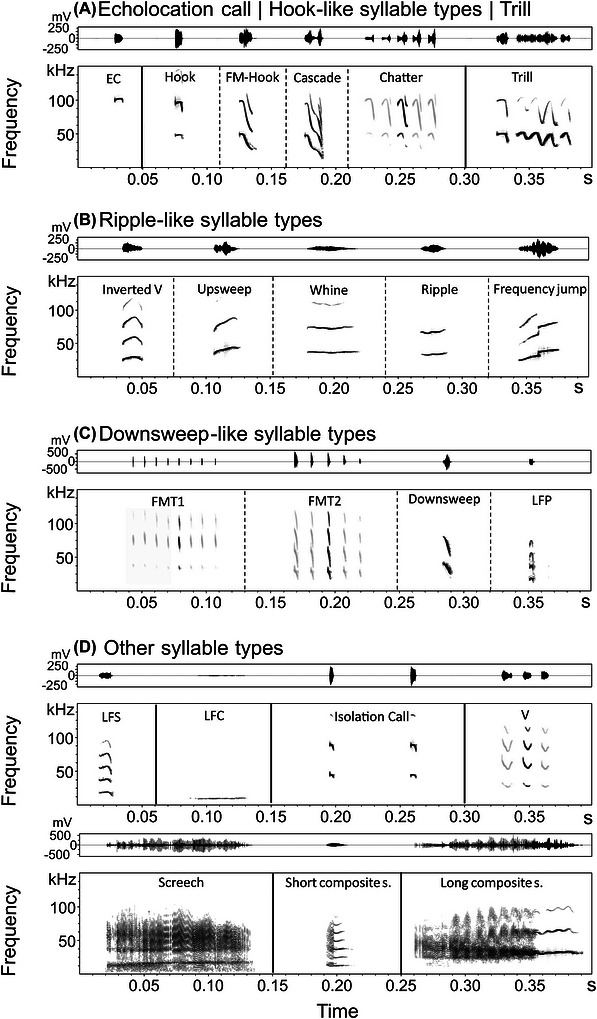
The 22 syllable types of *R. naso*. Spectrograms depict frequency over time, and oscillograms depict voltage over time. Syllable type classes are divided by solid lines, syllable types by dashed lines. Parameter used for creating the figure: FFT‐length = 1024, Frame size = 75%, window = Hamming. Abbreviations: FMT, frequency‐modulated train; LFC, low‐frequency call; LFP, low‐frequency pulse; LFS, low‐frequency syllable. Some parts of the spectrogram are lighter in color to focus attention on the single syllables, although they are often part of a multisyllabic vocalization.

Based on spectral similarity, the 22 syllable types were grouped into 12 syllable type classes (Figure 2). This classification was also supported by a DFA, which achieved 88.4% accuracy (range: 80.8%–100%), again significantly better than chance (9.1%; *n* = 681 syllables, binomial test: *p* < 0.001). Hook‐like syllables were characterized by a quasi‐constant or slightly upward‐modulated start, followed by a prominent downsweep. Their first harmonic began at a frequency higher than 45 kHz. Ripple‐like syllables were comparatively long and exhibited a quasi‐constant frequency curvature with small frequency modulation. Downsweep‐like syllables exhibited a strong negative frequency modulation, began with a first harmonic frequency of less than 45 kHz, and rarely featured an upward‐modulated or constant start.

Some syllables were produced singly (monosyllabic vocalization) or repeated in series (simple multisyllabic vocalizations), while others occurred only in combination with different syllables, forming complex multisyllabic vocalizations. Gradual transitions between vocalization types often occurred, for example, between the *V* and *FMT2*, or *Chatter* and *Trill*. The full vocal repertoire included four monosyllabic (*Whine, Frequency jump, Screech, Long composite syllable*), eight simple multisyllabic (*Chatter, Isolation call, FMT1, FMT2, LFC, Upsweep, Echolocation call, Hook*), and five complex multisyllabic vocalization types *(Complex Call with V train, Crescendo, Variable complex call, Pup vocal sequences, Song*).

The longest complex vocalization type is a *Song*, with a mean length ± SD of 3.0 ± 1.1 s (range: 0.9−6.0 s, *n* = 55) and on average ± SD 11.3 ± 2.2 syllable types (range: 7−16, *n* = 55) and 93.5 ± 37.4 syllables (range: 25−194, *n* = 55). While some syllable types (*Hook, Crescendo, FMT1*) appear outside of *Songs*, six syllable types (*Decrescendo, Dip, FM‐hook, Cascade, Downsweep*, and *Low frequency pulse)* are predominantly found in the *Song*. The *Song* is also one of the few vocalizations audible to humans, with a large bandwidth (mean: 34 kHz in the first harmonic) and a minimum frequency often reaching 15 kHz.

Proboscis bats produced four vocalization types in aggressive contexts, four during mother–pup interactions, and three during neutral behavior such as rocking (Figure 3). Four vocalization types could not be assigned to a specific context. The *Song* is male‐specific and primarily produced during the mating season. Echolocation calls were emitted in the day roost, for example, in case of potential danger. A typical example of this is a bird passing by the roost in close proximity. Pup‐specific calls included simple *Isolation calls*, produced a few minutes after birth, as well as complex *Pup vocal sequences* of varying structure. *Pup vocal sequences* resemble the *Song*, but are produced by juvenile bats, have higher intersyllable intervals, and fewer syllable types. They could be precursors of the *Song*, but this warrants further studies.

FIGURE 3The 17 vocalization types of *R. naso*. Spectrograms depict frequency over time, and oscillograms depict voltage over time. Vocalization types are divided by solid lines. The Upsweeps are shown with Hooks and Echolocation calls in the background. Low arousal vocalizations are produced mainly in a group context without aggressive interactions. Parameter used for creating the figure: FFT‐length = 1024, Frame size = 75%, window = Hamming.

**Figure:**
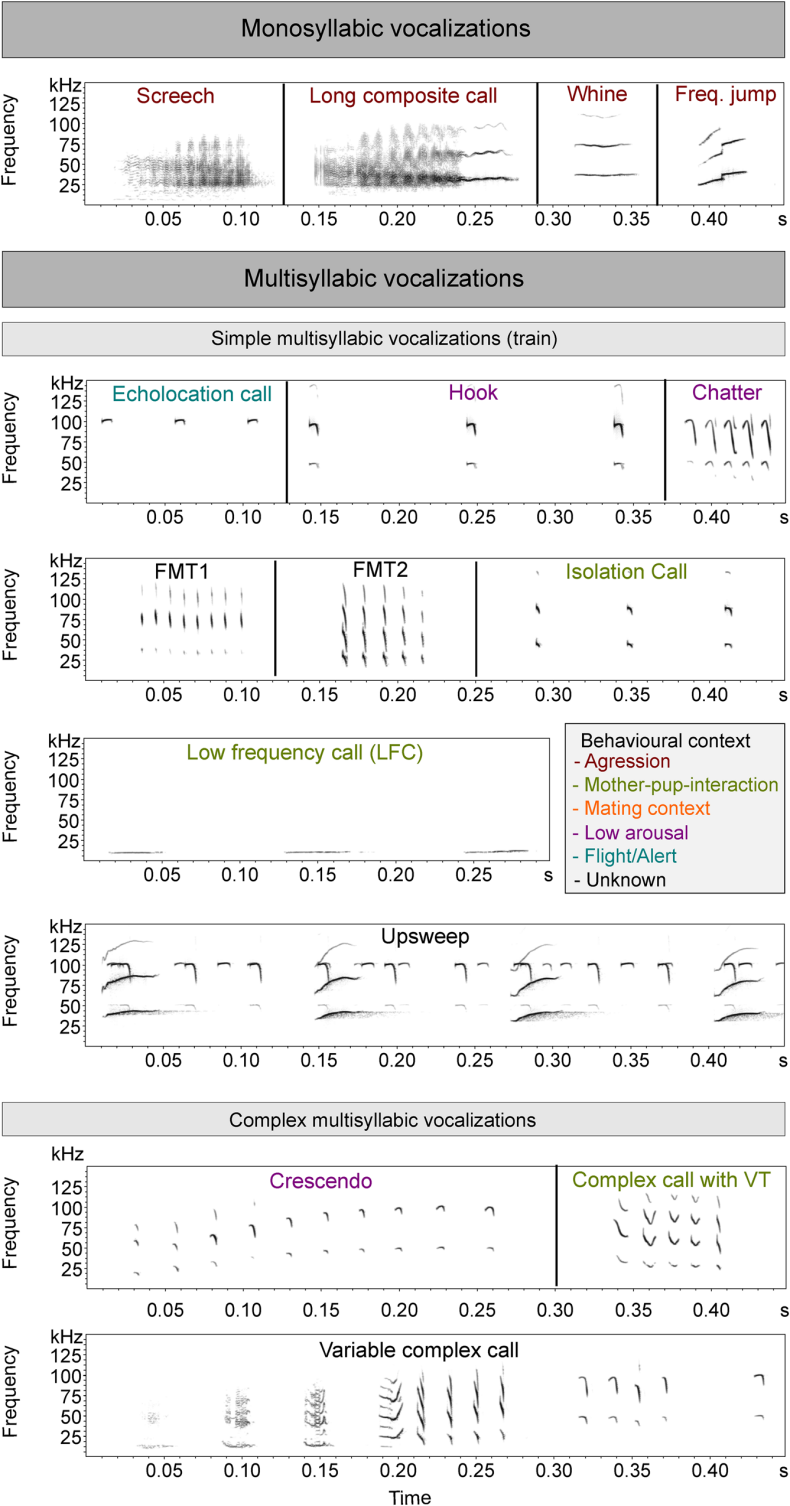

**Figure:**
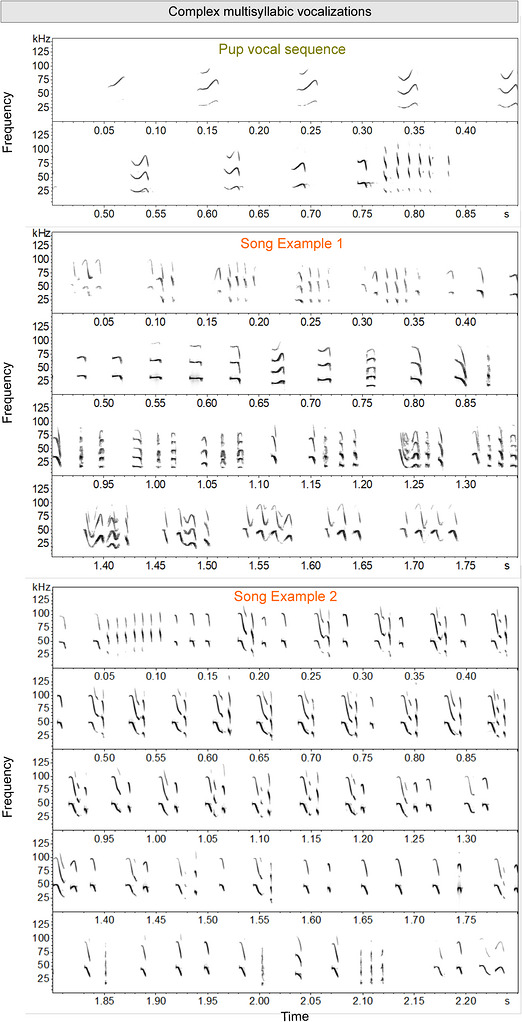

Detailed descriptions of all vocalization and syllable types, and their acoustic measurements, are provided in the Supplementary Materials (Results S1: descriptions and measurements, Tables S2 and S3). Here, we focus only on the most common one. *Hooks* are both a syllable type (when occurring in combination with other syllable types) and a vocalization type (when uttered in series) and are produced by individuals of all sexes. A *Hook* starts similarly to an *Echolocation call*, but features a more frequency‐modulated downsweep, reaching a peak end frequency of 84 kHz in its most dominant harmonic (H2). In some cases, the first harmonic (fundamental frequency) is undetectable. *Hooks* are often emitted in series with a regular interval of 53 ± 22 ms. As a vocalization type, *Hooks* are commonly produced during rocking behavior, before and during copulations, and while being vigilant in the night roosts. As a syllable, *Hooks* are frequently combined with *FMTs*, and occur in *Crescendos, Variable complex calls*, and *Songs*.

### 3.2 Personal Information Encoded in *Hooks*

The most common syllable and vocalization type, the *Hook*, encoded information on individual identity, sex, and colony affiliation. The acoustic parameters of *Hooks* from two different colonies exhibited significant differences between colonies (MANOVA; *F*_11,170_ = 26.218, *p*<0.001; partial η^2^ = 0.629). The largest colony differences were found for the distance to the maximum amplitude and the peak end frequency (Table 1). Males and females also differed significantly in acoustic parameters of *Hooks* (MANOVA; *F*_11,170_ = 5.123, *p*<0.001; partial η^2^ = 0.249), but these differences were much smaller than differences between colonies (Table 1).

**TABLE 1:** MANOVA results of the test for colony and sex information encoded in Hooks.

Acoustic parameters	Colony differences		Sex differences	
	*p*‐value	Partial eta squared	*p*‐value	Partial eta squared
Duration	**<0.001**	**0.082**	0.087	0.016
Interval	**0.010**	**0.036**	0.701	0.001
Distance to maximum amplitude	**<0.001**	**0.482**	0.887	0.000
Peak freq (start)	**<0.001**	**0.073**	**0.006**	**0.041**
Peak freq (end)	**<0.001**	**0.143**	**0.006**	**0.041**
Peak freq (maxpeakhold)	**<0.001**	**0.176**	**<0.001**	**0.094**
Freq curvature 1 (PC1)	**<0.001**	**0.096**	**0.001**	**0.058**
Freq curvature 2 (PC2)	**<0.001**	**0.119**	**0.028**	**0.027**
Freq curvature 3 (PC3)	**0.028**	**0.027**	**0.002**	**0.053**
Freq curvature 4 (PC4)	**0.003**	**0.049**	0.367	0.005
Freq curvature 5 (PC5)	0.107	0.014	0.217	0.008

*Note*: Significant *p*‐values (<0.05) are highlighted in bold.

A DFA (Table S10) performed on *Hooks* from six individuals revealed moderately strong individual differences in *Hooks*. 63.7% of *Hook* recordings were correctly classified to the respective individual (Table 2; chance level: 16.67%). This classification was significantly better than a random classification (binomial test, *p*<0.001) for four individuals.

**TABLE 2:** DFA results for individual information encoded in *Hooks*.

Bat ID	Predicted individual (%)						Number of recordings		
	A	B	C	D	E	F	Training set	Test set	Correctly classified
**A**	**64.7**	0	5.9	23.5	0	5.9	17	17	11
**B**	0	40	10	50	0	0	11	10	4
**C**	11.1	11.1	33.3	44.4	0	0	8	9	3
**D**	4.3	17.4	0	**78.3**	0	0	24	23	18
**E**	0	9.1	0	0	**81.8**	9.1	11	11	9
**F**	9.5	0	0	4.8	23.8	**61.9**	20	21	13

*Note*: The chance level of correct classification is 16.67%. Significantly better classification than random is highlighted in bold (*p* < 0.001).

*Hooks* closely resemble echolocation calls in key acoustic parameters such as duration, interval, peak, and maximum frequency (Table S11). In the DFA, the acoustic centroids of *Hooks* and *Echolocation calls* were more similar to each other than the average pairwise distance between other syllable types within the first three discriminant functions (3.0 vs. 6.2). This suggests that *Hooks* may also convey echo‐acoustic information about the environment. This potential dual function supports the hypothesis that *R. naso* reduce the need for conspicuous signaling.

### 3.3 High‐Frequency Communication as an Adaptation to Crypsis

**FIGURE 4:**
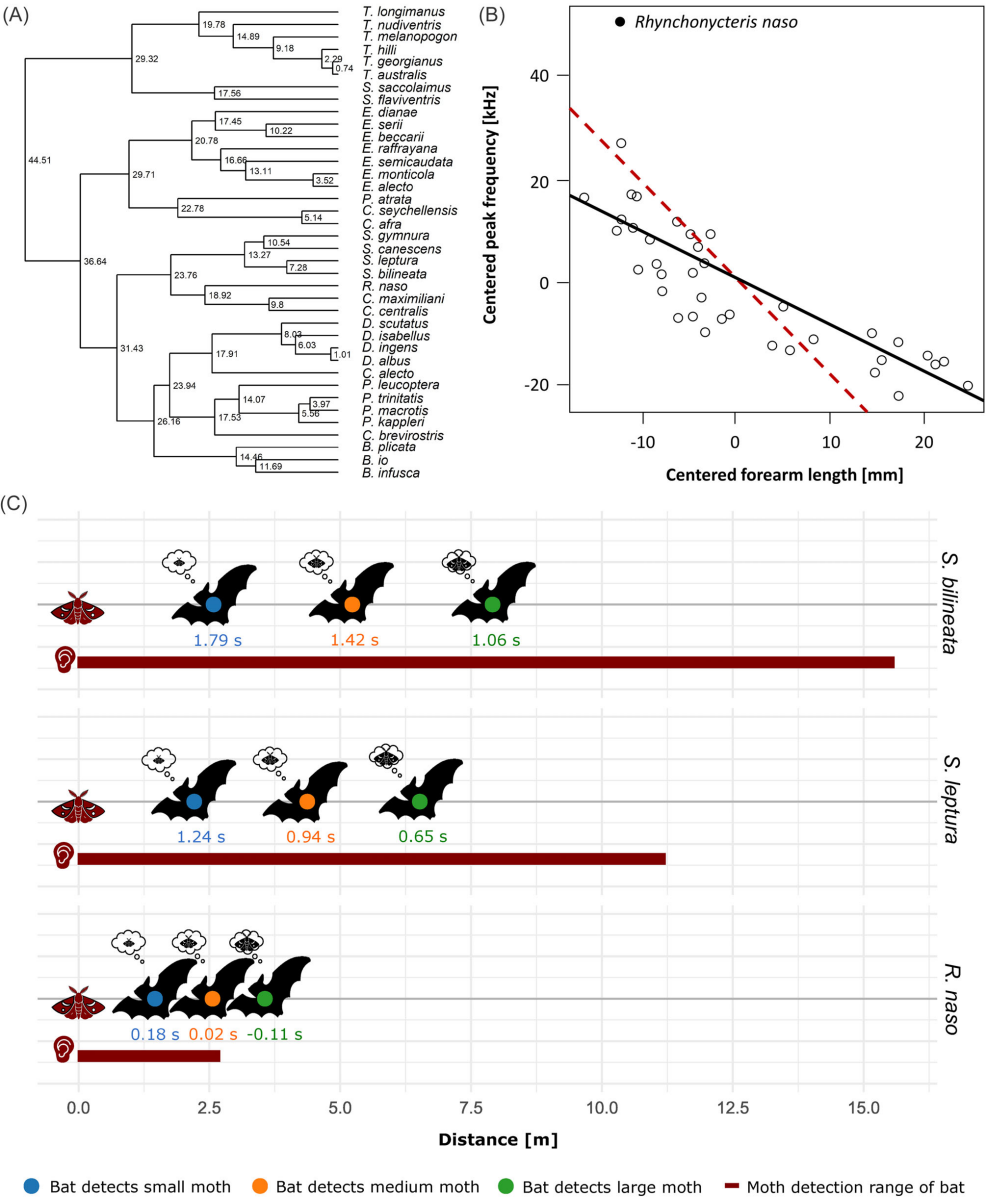
Echolocation analysis. (A) The phylogenetic tree of emballonurid bats used for the SLOUCH analysis was adapted from [42], and numbers show divergence times in millions of years. (B) The optimal regression slope (red) describes the expected relationship between body size and peak end frequency of echolocation calls if no constraints on the evolution toward the optimal state existed (i.e., phylogenetic inertia). The evolutionary regression slope (black) depicts the current relationship between body size and peak end frequency of echolocation calls. (C) Detection distances and escape times for interactions between bats and eared moths. Colored dots represent distances at which bats detect moths of different sizes (blue = small, orange = medium, green = large), and red bars show the detection range of moths. In *R*. *naso*, bats and moths detect each other almost simultaneously. In sympatric bat species, moths detect the bats consistently earlier. Reported time spans indicate the times moths have to escape before they are detected by bats. Negative times refer to situations where bats detect the moths before they are detected themselves, assuming a flight speed of 7.3 m/s for all three bat species.

We hypothesized that the vocal repertoire is dominated by high‐frequency syllables, which attenuate quickly and fall outside most predators’ and prey's hearing range, thus enabling inconspicuous echolocating and social communication. To test whether the unusually high echolocation call frequency of *R. naso* (∼100 kHz) aligns with allometric expectations based on body size, we applied a phylogenetic comparative approach across 38 emballonurid bats. We tested the relationship between mean forearm length, as a proxy for body size, and the peak frequency at the end of echolocation calls using a phylogenetically controlled analysis. We detected a negative relationship between body size and signal frequency (slope = −1.85 ± 0.38, *R* ^2^ = 0.38, AICc = 278.7, logL = −134.8), indicating that an evolutionary increase in body size was associated with a decrease in signal frequency and vice versa (Figure 4 and Table S4). *R*. *naso* echolocates at a much higher frequency than expected by body size and phylogeny alone, suggesting an adaptation to reduce detectability by predators or prey.

Because *R. naso* uses high‐frequency echolocation, prey arthropods are detected only at short distances, ranging from 1.5 to 3.6 m, depending on prey size and echo strength. These detection distances are substantially shorter than those of closely related, sympatric, lower‐frequency bat species (e.g., 2.6–7.9 m in *S. bilineata* and 2.2–6.5 m in *S. leptura*). However, eared moths, despite their sensitivity to ultrasound, detect *R. naso* only at similarly short distances (<2.7 m, varying with environmental conditions). As a result, *R. naso* and moths detect each other almost simultaneously, leaving moths little time to initiate evasive responses. In contrast, moths detect approaching sympatric bat species at much greater distances (11.3 m for *S. leptura* and 15.6 m for *S. bilineata*), typically before being detected by the bats, resulting in substantially longer escape times (e.g., 1.2 and 1.8 s for a small moth escaping *S. leptura* and *S. bilineata*, respectively; Figure 4C). These findings highlight that the high‐frequency echolocation calls of *R. naso* substantially reduce the typical detection asymmetry between bats and eared moths and may enhance capture success, in line with acoustic crypsis.

**FIGURE 5:**
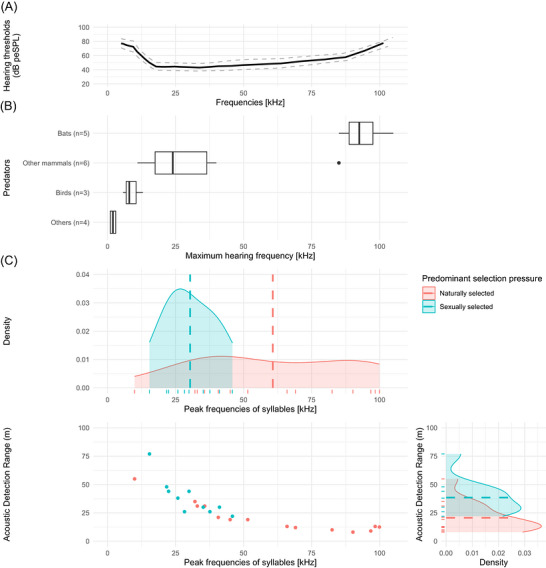
Distribution of peak frequencies in the vocal repertoire of *R. naso* and their relation to hearing thresholds and acoustic detection ranges. (A) Hearing threshold of proboscis bats, modified after [97]. (B) Potential predators of proboscis bats and their maximum hearing frequencies. The single dot in “Other mammals” are domesticated cats, but also other nondomesticated felids such as jaguarundis can hear up to 65 kHz. (C) Scatterplot depicting the relation between acoustic detection range and peak frequencies of syllable types. Bottom and right: Histograms of these datapoints, grouped with respect to their predominant selection pressures, are shown smoothed in density plots. dashed line = mean, small lines under histograms visualize datapoints. These are identical to the scatterplot. Details are provided in Tables S11 and S12.

To investigate additional factors that may shape syllable frequencies, we assessed the hearing sensitivities of proboscis bats and their predators, as well as the selection pressures acting on different syllable types. The vocal repertoire of *R. naso* consists mostly of pure tonal syllables, which are generally higher in frequency than noisy ones. Proboscis bats’ syllables span a broad frequency range (9−100 kHz, Figure 5C, upper graph), closely matching the species’ hearing sensitivity (Figure 5A). In contrast, most predators of proboscis bats cannot hear above 40 kHz and, therefore, miss a large portion of these syllables. Notable exceptions are other echolocating mammals (bats) and felids, which can perceive higher frequencies (Figure 5B and Table S12). The peak frequencies of syllables appeared to depend on the selection pressure shaping them the most. Predominantly naturally selected syllables covered the full frequency span (9−100 kHz), with only five syllables below 40 kHz (mean peak frequency = 60.7 ± 29.4 kHz). This affects their acoustic detection range, which we estimated based on both peak frequency and estimated syllable amplitude. Naturally selected syllables were significantly less detectable over distance compared to sexually selected ones, with a mean detection range of 20.6 ± 13.2 m (*n* = 14) versus 38.5 ± 16.2 m (*n* = 10; Wilcoxon rank sum test: *W* = 24, *p* < 0.01). Predominantly sexually selected syllables clustered in a narrower frequency band (15–45 kHz) and had significantly lower peak frequencies with an average of 30.4 ± 9.5 kHz (Welch‐Test, *t* = 3.61, df = 16.6, *p*‐value < 0.01). This caused their greater propagation distance (Figure 5C, bottom and Table S12). In summary, naturally selected syllables in *R. naso* tended to be high in frequency and short‐ranged: features consistent with an adaptive strategy for remaining cryptic during vocal production.

### 3.4 Timing of Vocalizations—Crypsis versus Attention

**FIGURE 6:**
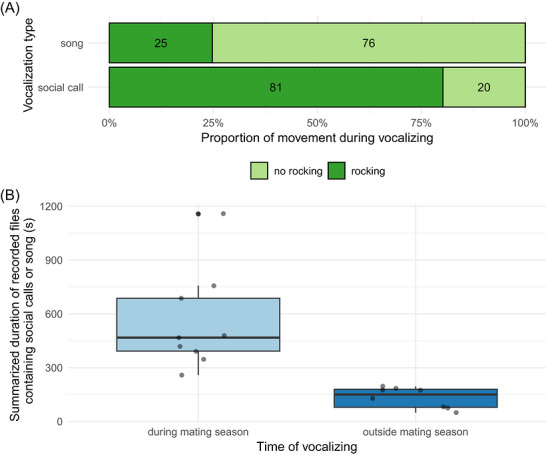
Timing of the vocalization. (A) Proportion of movement during vocalizing. The numbers inside the bars represent the sample size of vocalizations. (B) Comparison of the sum of the duration of audio files with social vocalizations during and outside the mating season.

We hypothesized that, in addition to frequency adaptations of the vocal repertoire, proboscis bats may shift their vocal activity to rocking periods to reduce detectability by unintended receivers. In contrast, we expected sexually selected vocalizations (such as *Song*) to deviate from this pattern, as selection should favor more conspicuous signals in this context. Various social calls were predominantly uttered during rocking periods (percentage during rocking = 80%, *n* = 101 social calls), including isolation calls of pups. However, *Song* was predominantly produced during periods without rocking behavior (percentage during rocking = 25%; *n* = 101 *Songs*, Figure 6A). We also compared the amount of social calling and singing during the mating season and outside the mating season. Proboscis bats show a strong seasonal pattern, with significantly higher vocal activity (social calls and songs combined) during than outside the mating seasons (Wilcoxon test, *t* = 4.44, df  =  8.79, *p* < 0.01, *n* = 8, Figure 6B). These results indicate that *R. naso* adjust the timing of the vocalizations depending on their current needs, to reduce predation pressure and/or to ensure mating success.

## 4 Discussion

Proboscis bats have a large vocal repertoire and adaptations to communicate vocally while remaining cryptic. One common social call resembles a modified echolocation call, suggesting an additional sensory function in addition to its social function. The majority of their vocalizations are characterized by high frequencies and short detection ranges, and are produced at specific timepoints when detection from heterospecifics is minimal. In contrast, sexually selected vocalizations are more conspicuous. In addition, *R. naso* emits exceptionally high‐frequency echolocation calls that exceed allometric expectations, consistent with acoustic crypsis.

The large vocal repertoire of proboscis bats may be linked to their complex social life. The social complexity hypothesis suggests that cognitively demanding tasks such as individual recognition and hierarchy formation promote vocal complexity across animal taxa, including bats [53]. Although their group sizes are moderate, proboscis bats live in stable, mixed‐sex groups with frequent interactions, nonrandom associations, and shifting male hierarchies tied to roost sites [40, 54]. These socially diverse groups likely require flexible communication, supporting the need for a rich vocal repertoire.

Approximately 30 vocal repertoires of bat species have been described, representing less than 3% of all bat species. Many studies focus on specific vocalizations (pup isolation calls, aggressive calls, songs), sex or age of the caller [55, 56, 57, 58, 59]. Undoubtedly, there are differences in the complexity of vocal repertoires between species. However, it can be difficult to compare vocal repertoire studies directly. There are different definitions for vocal repertoires (vocalizations or syllables), different recording situations (devices, laboratory, or field conditions), and interpretations of how the data is categorized. Nevertheless, we conclude that proboscis bats have a vocal repertoire that is comparable in size to that of *S*. *bilineata*, another emballonurid bat that is well‐studied [60, 61, 62].

We found moderate group, sex, and individual signatures in the most common social call, the *Hook*. This suggests that *Hooks* could help group members identify which individual perches within a social group without moving conspicuously. Individual signatures have also been found in many other bat vocalizations, including echolocation calls [63], mother–infant‐mediating calls [55, 64, 65, 66, 67], and courtship vocalizations [68, 69]. It is likely that other vocalizations of proboscis bats, such as *Isolation calls* or male *Song*, also encode individual signatures. *Hooks* are structurally very similar to echolocation calls and may have a dual function of communication and echolocation, which has been documented in multiple bat species [63, 70, 71]. *Hooks* are often produced during copulations, which usually occur outside of rocking periods, that is, at times when visual protection is lower. The ability to communicate and scan the environment simultaneously is efficient and may serve as an additional antipredator adaptation.

*R*. *naso* emits unusually high‐frequency echolocation calls (∼100 kHz), and different hypotheses have been proposed to explain this phenomenon. The allometry hypothesis links smaller body size to higher call frequencies, and a negative correlation between body mass and frequency has been found in bats [72]. While our data support this trend, *R. naso* still exceeds frequency expectations based on forearm length, even after correcting for phylogeny. The foraging habitat hypothesis posits that high frequencies are advantageous in cluttered environments [73], but *R. naso* forages over open water [36], and other species foraging similarly use lower frequencies [74]. According to the prey‐detection hypothesis, high frequencies enhance detection of small prey [73, 75, 76], and the acoustic communication hypothesis suggests frequency shifts serve to minimize overlap with other species, promoting acoustic niche separation [73, 77, 78].

Our results support the allotonic frequency hypothesis, which argues that high‐frequency calls reduce detection by hearing insects [25, 73, 79]. Our results show that echolocation calls of *R. naso* remain undetected by hearing moths until very short distances (∼2.7 m), resulting in near‐simultaneous detection of predator and prey. This minimizes the escape time of moths and likely increases capture success. Although *R. naso* mainly feeds on nonhearing aquatic emergent insects, such as Diptera, especially chironomid midges [80, 81, 82], its diet also includes arthropods from the orders Hemiptera, Coleoptera, Lepidoptera, and Orthoptera [79, 80, 82]. Notably, *R. naso* feeds on at least five hearing Lepidoptera families [79]. While members of the Erebidae [51], Nymphalidae [83, 84, 85], and Sphingidae [86] can hear well up to 40 kHz, members of Geometridae [50] and Noctuidae [50, 51] can hear well up to 90 kHz, making them difficult prey for bats. However, closely related *S. bilineata* also exploit six hearing lepidopteran families, and *S. leptura* and *Rhogeessa aeneus* feed on arthropods, which can hear up to 95 kHz, despite not using such high‐frequency echolocation [82, 87, 88, 89]. This suggests that such prey can be captured without relying on high‐frequency echolocation. However, given that *R. naso* appears specialized on emerging aquatic insects, which can show substantial temporal variability due to environmental and hydrological conditions [90, 91], the ability to reliably forage on hearing prey may provide a key selective advantage.

We further propose a novel, additional explanation for the high frequency of *R. naso's* echolocation calls: acoustic crypsis to lessen detection by predators. Supporting this, *R. naso* also emits quieter echolocation calls than its close relative *S. bilineata* during both search and buzz phases [47], suggesting further adaptations for remaining inconspicuous. Taken together, our findings support the hypothesis that high‐frequency echolocation calls reduce detection by prey, and we also extend this hypothesis by suggesting reduced detectability by predators.

Acoustic crypsis appears to have also shaped the social vocalizations of *R. naso*. Naturally selected syllables had significantly higher peak frequencies and shorter acoustic detection ranges than sexually selected ones, thus supporting our hypothesis of acoustic crypsis in *R. naso*. We suggest that this frequency shift reflects an adaptation to avoid detection by predators, most of which have limited hearing abilities and do not perceive sounds above 40 kHz. As a result, many naturally selected syllables are likely inaudible to them. Although some predators, such as carnivorous bats and felines, are capable of detecting higher frequencies, they are mostly active during dusk, night, and dawn. During these times, *R. naso* is more mobile, less socially vocal, and less reliant on crypsis. This likely reduces the selective pressure from these exceptional predators.

We calculated the active space of *R. naso* syllables and found that it is relatively short, approximately 20 m for naturally selected and 39 m for sexually selected syllables. This limited range may reflect an antipredator adaptation, as short detection distances reduce the risk of being overheard. In contrast, other bat species such as *S. bilineata* [92]*, Phyllostomus hastatus* [93], and *Cardioderma cor* [94] produce vocalizations with detection distances exceeding 100 m. Detection distances were estimated based on the average hearing threshold of bats (–20 dB detection threshold [48]). Since most predators have poorer high‐frequency hearing than bats, the actual detection distance from a predator's perspective is likely shorter. Environmental and contextual factors may further reduce the actual detection distances. *R. naso* often roosts in vegetation and always close to streams [36, 81, 95], which are acoustically cluttered and noisy environments. Vegetation scatters and absorbs sound, especially high frequencies, while background noise from flowing water, insects like cicadas, and wind can mask vocalizations. Additionally, fluctuations in temperature and humidity influence how well sound travels through the air. Taken together, it is likely that our detection distances overestimate how far syllables travel under natural conditions.

As expected, sexually selected syllables reached farther than naturally selected ones. This aligns with the idea that sexually selected signals may serve to advertise traits such as body size or aggressive intent, and may need to reach conspecifics flying nearby, distant group members, or even neighboring colonies. It is currently unknown whether long‐distance communication occurs in *R. naso*, for example, between neighboring social groups or to attract females or deter nongroup males. However, because some of our study colonies were only a few meters apart (10−20 m), it seems likely that low‐frequency vocalizations (like the *Song*) are perceived in neighboring colonies. Although our study focused on day roosts, male *Song* has occasionally been recorded at night roosts (L. Dressler, unpublished data), and copulations also commonly occur at night [40]. Future studies could, therefore, test whether nocturnal *Song* may serve in mate attraction. In closely related *S. bilineata*, low‐frequency territorial songs allow males to attract dispersing females searching for new colonies from distances of over 100 m [92].

Proboscis bats shifted their vocal activity to specific periods to reduce detectability by unintended receivers, but sexually selected vocalizations (such as *Song*) deviated from this pattern. Most social vocalizations were produced during the mating season, and *Song* was produced only then, potentially reflecting an increased motivation to communicate and a higher benefit for reproductive success that outweighs predation risk. Naturally selected vocalization types were typically uttered during rocking events. Rocking is an antipredator behavior of proboscis bats, associated with wind gusts, that allows them to remain cryptic while in motion [38, 96]. Thus, our results extend the list of behaviors (e.g., stretching, grooming, urinating [38]) that remain more cryptic during rocking to include vocalizing, providing behavioral evidence for a link between acoustic signaling and crypsis. Increased vocal activity during rocking may have multiple explanations: the behavioral synchrony may promote more social interactions, increasing the need to communicate; the bats may be confident that group members are awake and attentive; and the wind noise may mask their calls from eavesdropping predators. This makes it more difficult to locate the signaler. In contrast, *Songs* are typically emitted during silent periods. They may be directed at distant conspecifics and potentially benefit from reduced environmental noise, for example, during times without wind, allowing them to travel further. This could help attract potential mates or deter rivals. *Songs* may function as fitness indicators, as they are typically loud, low‐frequency, and long. These features make the signal highly conspicuous, increasing the risk of detection by predators. Sexually selected vocalizations are, therefore, most likely shaped by selection for effective long‐distance communication, while increased detectability by predators represents a secondary cost rather than a primary driver. Notably, however, the risk is not limited to the singing male; the entire group may become more vulnerable during such conspicuous displays.

In addition, the high‐frequency nature of social vocalizations may reflect shared vocal anatomy and physiology with echolocation calls, suggesting these traits have not evolved independently but are constrained by common mechanisms of sound production and perception. More generally, the vocal repertoire of *R. naso* may stem from a coevolutionary interplay between foraging and roosting ecology. High‐frequency echolocation may initially have evolved in the context of prey detection and subsequently influenced the evolution of social vocalizations, possibly facilitating the use of very exposed roosting sites. Alternatively, selection for reduced detectability in exposed roosting sites may have favored higher vocalization frequencies.

Future studies could include playback experiments to investigate the functional context of vocalizations and refine syllable classification. It would also be valuable to test behavioral responses under experimentally increased predation risk, for instance, by presenting predator and nonpredator sounds. This would help to explore the weight of predator detection on vocal behavior in *R. naso*.

## 5 Conclusion

Our study reveals that *R. naso* resolves the tension between communication and concealment through a diverse set of vocal adaptations consistent with acoustic crypsis. Its exceptionally high‐frequency echolocation calls suggest that acoustic crypsis extends beyond social signaling into orientation and foraging. Most naturally selected vocalizations are structured and timed to reduce detection by predators and prey, whereas sexually selected signals are more conspicuous, indicating that sexual selection can override pressures for crypsis. Together, these findings show that the vocal system of *R. naso* has been shaped by the interacting forces of social complexity, foraging ecology, predator avoidance, and sexual selection, highlighting this species as a powerful model for understanding how multiple selective pressures drive the evolution of animal communication.

## Author Contributions

Conceptualization: M.K. and M.N.; Methodology: L.E.D., K.‐H.F., M.K., and M.N.; Data acquisition: L.E.D., M.K., and M.N.; Formal analysis and investigation: L.E.D., K.‐H.F., M.K., and M.N.; Writing – original draft preparation: L.E.D.; Writing – review and editing: L.E.D., K.‐H.F., M.K., and M.N.; Funding acquisition: L.E.D., M.K., and M.N.; Resources: M.K.; Supervision: M.K. and M.N.

## Conflicts of Interest

There are no conflicts of interest.

## Supporting information




**Supplementary Materials**: nyas70345‐sup‐0001‐SuppMat.docx

## Data Availability

Data available in the article's Supplementary Materials.
