# Supplementary material for: Cryptic Vocal Communication in the Proboscis Bat (Rhynchonycteris naso)
Source: Ann N Y Acad Sci. 2026 Jul 21;1561(1):e70345. doi: 10.1111/nyas.70345 (PMC13387082; doi:10.1111/nyas.70345)
Supplement: Supplementary file 1 — Supplementary Materials: nyas70345‐sup‐0001‐SuppMat.docx [file NYAS-1561-0-s001.docx]

Supporting Information for “Cryptic Vocal Communication in the Proboscis Bat (*Rhynchonycteris naso*)”

Lena E. Dressler^1,2^, Karl-Heinz Frommolt^1^, Mirjam Knörnschild^1,3,4*^, Martina Nagy^1,*^

Affiliations:

1. Museum für Naturkunde, Leibniz Institute for Evolution and Biodiversity Science, Berlin, Germany
2. Freie Universität Berlin, Institute for Biology, Berlin, Germany
3. Humboldt-Universität zu Berlin, Institute for Biology, Berlin, Germany

4) Smithsonian Tropical Research Institute, Balboa, Panama

*) joint last authors

corresponding author: Lena E. Dressler (lena.dressler@mfn.berlin), ORCID:0000-0003-4579-1152

Contents

[Supporting Results 1 3](#_Toc227439265)

[Syllable type descriptions 12](#_Toc227439266)

[Vocalization type descriptions 13](#_Toc227439267)

[Monosyllabic vocalizations 13](#_Toc227439268)

[Multisyllabic simple vocalizations/trains 14](#_Toc227439269)

[Multisyllabic complex vocalizations 16](#_Toc227439270)

Tables

[**Table S1:** Colony size per recording season in Guanacaste, Costa Rica. 3](#_Toc227439243)

[**Table S2:** Overview of syllable types and their classes of Proboscis bats and their main acoustic measurements. 4](#_Toc227439244)

[**Table S3:** Overview of vocalization types of Proboscis bats 5](#_Toc227439245)

[**Table S4:** Echolocation call peak frequencies and mean forearm length of Emballonurid species used in the SLOUCH analysis. 6](#_Toc227439246)

[**Table S5:** Frequencies and amplitudes per syllables type needed for acoustic range calculation. 9](#_Toc227439247)

[**Table S6:** DFA classification success for tonal syllable types. 10](#_Toc227439248)

[**Table S7:** Assessment of model fit for the DFA of tonal syllables. 11](#_Toc227439249)

[**Table S8:** DFA classification success for non-tonal syllable types. 11](#_Toc227439250)

[**Table S9:** Assessment of model fit for the DFA of non-tonal syllables. 11](#_Toc227439251)

[**Table S10:** Results of the DFA for testing of individual information encoded in the vocalization type *Hook.* 18](#_Toc227439252)

[**Table S11:** Comparison between *Echolocation call* syllables, *Hook* syllables and average tonal syllables of *R. naso*. 18](#_Toc227439253)

[**Table S12:** Potential predators and prey of Proboscis bats and their maximum hearing frequency. 19](#_Toc227439254)

## Supporting Results 1

**Table S1:** Colony size per recording season in Guanacaste, Costa Rica.

| Col. | age | Mar./Apr. 2022 | Oct./Nov. 2022 | Mar./Apr. 2023 | Oct./Nov. 2023 | Average |
| --- | --- | --- | --- | --- | --- | --- |
| 1 | AD | 52.94 ± 5.22 | 40.84 ± 5.73 | 42.96 ± 4.63 | 47.46 ± 6.81 | 46.05 ± 4.64 |
|  | pup | 0 - 9 | 0 - 9 | 0 - 16 | 0 - 13 | 0 - 16 |
| 2 | AD | 20.00 ± 3.71 | 20.35 ± 1.93 | 15.06 ± 1.12 | 14.16 ± 2.33 | 17.39 ± 2.80 |
|  | pup | 0 - 1 | 1 - 9 | 0 - 6 | 0 - 4 | 0 - 9 |
| 3 | AD | 12.69 ± 4.62 | 11.41 ± 1.44 | 14.15 ± 7.29 | 9.42 ± 1.26 | 11.92 ± 1.73 |
|  | pup | 0 - 5 | 0 - 1 | 0 - 5 | 0 - 3 | 0 - 5 |
| 4 | AD | 14.57 ± 4.69 | 21.35 ± 6.15 | 15.72 ± 5.74 | 21.00 ± 3.95 | 18.16 ± 3.04 |
|  | pup | 0 - 7 | 0 - 7 | 0 - 7 | 0 - 7 | 0 - 7 |
| 5 | AD | 11.20 ± 2.82 | 8.33 ± 3.14 | 9.80 ± 1.35 | 9.13 ± 3.31 | 9.62 ± 1.05 |
|  | pup | 0 - 4 | 0 - 1 | 0 - 5 | 0 - 2 | 0 - 5 |

Col = colony, AD = mean number and SD of adults, pup = minimum and maximum number of pups and juveniles present during the recording seasons

**Table S2:** Overview of syllable types and their classes of Proboscis bats and their main acoustic measurements.

| Syllable type class | Syllable type | Entropy | Dominant harmonic | Duration (ms) | Peak frequency  (1st Harmonic, kHz) | Bandwidth  (1st Harmonic, kHz) | N |
| --- | --- | --- | --- | --- | --- | --- | --- |
| Hook-like | *Hook* | tonal | 2 | 5.21 ± 1.60 | 48.28 ± 1.03 | 8.79 ± 2.37 | 28 |
|  | *FM hook* | tonal | 1 or 2 | 7.05 ± 1.77 | 42.79 ± 3.12 | 22.57 ± 3.57 | 25 |
|  | *Cascade* | tonal | 1 or 2 | 13.68 ± 2.33 | 31.63 ± 3.66 | 31.15 ± 4.98 | 24 |
|  | *Chatter* | tonal | 1 or 2 | 6.5 ± 1.39 | 46.81 ± 2.46 | 14.36 ± 3.93 | 29 |
| Echolocation call | *Echolocation call* | tonal | 2 | 7.50 ± 1.54 | 49.80 ± 915 | 2.66 ± 0.34 | 29 |
| Trill | *Trill* | tonal | 1 or 2 | 46.62 ± 10.65 | 43.75 ± 4.30 | 20.56 ± 8.19 | 22 |
| Isolation call | *Isolation call* | tonal | 2 | 5.28 ± 1.21 | 40.94 ± 3.02 | 4.49 ± 1.24 | 25 |
| Downsweep-like | *FMT1* | tonal | 2 | 1.06 ± 0.29 | 34.43 ± 2.73 | 8.43 ± 1.39 | 24 |
|  | *FMT2* | tonal | 2 | 3.36 ± 1.36 | 25.57 ± 2.75 | 9.10 ± 1.67 | 48 |
|  | *Downsweep* | tonal | 1 or 2 | 3.63 ± 1.56 | 36.32 ± 2.40 | 13.48 ± 2.90 | 25 |
|  | *LFP* | tonal | 1 | 2.03 ± 1.00 | 22.25 ± 5.20 | 8.33 ± 2.65 | 49 |
| V | *V* | tonal | 1 | 9.68 ± 2.82 | 21.55 ± 4.81 | 9.19 ± 2.59 | 52 |
| Ripple-like | *Ripple* | tonal | 1 or 2 | 27.11 ± 21.35 | 31.28 ± 9.23 | 5.05 ± 1.50 | 58 |
|  | *Whine* | tonal | 1 | 45.36 ± 18.53 | 41.45 ± 4.64 | 3.20 ± 0.750 | 25 |
|  | *Upsweep* | tonal | 1 or 2 | 21.72 ± 8.37 | 35.46 ± 4.15 | 7.84 ± 2.50 | 38 |
|  | *Inverted V* | tonal | 1 or 2 | 13.24 ± 3.17 | 31.48 ± 5.85 | 7.17 ± 1.14 | 39 |
|  | *Frequency jump* | tonal | 1 | 23.96 ± 10.59 | 31.32 ± 3.47 | 12.15 ± 2.68 | 26 |
| LFC | *LFC* | tonal | 1 or 2 | 24.66 ± 10.08 | 9.90 ± 2.08 | 4.66 ± 1.41 | 23 |
| LFS | *LFS* | tonal | 1 | 9.12 ± 2.93 | 15.12 ± 2.14 | 5.66 ± 2.16 | 21 |
| Screech | *Screech* | noisy | no clear harmonics | 74.78 ± 26.39 | 28.13 ± 7.18 | 80.72 ± 9.96 | 24 |
| Long composite s. | *Long composite s.* | composite | no clear harmonics | 64.64 ± 25.86 | 35.83 ± 11.31 | 64.69 ± 21.65 | 24 |
| Short composite s. | *Short composite s.* | composite | no clear harmonics | 14.97 ± 2.94 | 27.16 ± 5.24 | 74.15 ± 7.84 | 23 |

**Table S3:** Overview of vocalization types of Proboscis bats

| **vocalization type** | **production mode** | **syllable type** | **behavioral context** | **frequency of  occurence** | **sex and age of  vocalising bat** | **can be part of** | **peak frequency  (all hamonics, kHz)** | **dominant  harmonic** |
| --- | --- | --- | --- | --- | --- | --- | --- | --- |
| *Echolocation call* | in series | monosyllabic | flight, alert, danger | very common | all bats | - | 99.38 ± 1.74 | 2 |
| *Hook* | in series | monosyllabic | rocking, copulation, during night | Ubiquitous | all adult bats | song, crescendo, decrescendo,  variable complex call, in combination with FMT | 88.35 ± 15.52 | 2 |
| *Chatter* | singly or in series | multisyllabic (train) | rocking behaviour,  before flight relocation | very common | all adult bats | song, variable complex calls | 70.61 ± 18.30 | 1 or 2 |
| *Upsweep* | singly | multisyllabic (train) | unknown context | common | all adult bats | song, crescendo, decrescendo | 50.22 ± 16.40 | 1 or 2 |
| *Whine* | singly | multisyllabic (train) | aggressive interaction | rare | all adult bats | - | 51.39 ± 15.89 | 1 |
| *Frequency jump* | singly | multisyllabic (train) | aggressive interaction | rare | all adult bats | - | 3.44 ± 4.94 | 1 |
| *Screech* | mainly in series | monosyllabic | aggressive interaction | very common | all adult bats | - | 28.13 ± 7.03 | no clear harmonics |
| *Long composite call* | mainly in series | monosyllabic | aggressive interaction | common | all adult bats | - | 33.47 ± 9.68 | no clear harmonics |
| *FMT1* | singly | multisyllabic (train) | unknown context | common | all adult bats | song, variable complex calls | 75.96 ± 20.01 | 2 |
| *FMT2* | singly | multisyllabic (train) | unknown context | common | all adult bats | song, variable complex calls | 5511 ± 28.73 | 2 |
| *LFC* | in series | multisyllabic (train) | mother-pup-interaction | rare | only weaning mothers | - | 11.64 ± 2.02 | 1 or 2 |
| *Isolation call* | singly or in series | multisyllabic (train) | mother-pup-interaction | common | pups of both sexes | - | 70.22 ± 28.17 | 2 |
| *Pup vocal sequences* | mainly in series | multisyllabic (complex) | mother-pup-interaction | rare | pups of unknown sexes | - | 34.50 ± 10.00 | 1 or 2 |
| *Complex call with V train* | singly | multisyllabic (complex) | mother-pup-interaction | common | pups of unknown sexes | V train in song | 37.97 ± 16.06 | 1 or 2 |
| *Crescendo* | singly | multisyllabic (complex) | rocking behaviour | very common | all adult bats | song | 62.20 ± 33.38 | 1 or 2 |
| *Variable complex call* | singly | multisyllabic (complex) | unknown context | common | all adult bats | - | 46.04 ± 15.11 | 1 or 2 |
| *Song* | singly or in series | multisyllabic (complex) | mating season, mostly in evening | rare | only adult male bats | - | 43.36 ± 9.32 | 1 or 2 |

**Table S4:** Echolocation call peak frequencies and mean forearm length of Emballonurid species used in the SLOUCH analysis.

| Species | Echol.  peak freq.  (kHz) | forearm  length  (mm) | source (forearm length) |
| --- | --- | --- | --- |
| *Balantiopteryx infusca* | 56.0 | 39.0 | Arroyo-Cabrales and Jones 1988; Koopman 1994 |
| *Balantiopteryx io* | 49.0 | 37.5 | Reid 1997; Arroyo-Cabrales and Jones 1988; Koopman 1994 |
| *Balantiopteryx plicata* | 40.6 | 42.2 | Arroyo-Cabrales and Jones 1988; Reid 1997; Koopman 1994 |
| *Centronycteris centralis* | 42.7 | 46.8 | Hice and Solari 2002; Woodman 2003; Simmons and Handley 1998 |
| *Centronycteris maximiliani* | 40.8 | 45.6 | Reid 1997; Hice and Solari 2002; Koopman 1994 |
| *Coleura afra* | 32.9 | 49.5 | Dunlop 1997; Thomas 1915; Koopman 1994 |
| *Coleura seychellensis* | *34.5* | 55.3 | Thomas 1915; Karl F. Koopman 1994 |
| *Cormura brevirostris* | 29.4 | 46.9 | Bernard 2003; Reid 1997; Koopman 1994 |
| *Cyttarops alecto* | 36.0 | 46.5 | Starrett 1972; Reid 1997; Koopman 1994 |
| *Diclidurus albus* | 24.0 | 65.8 | Ceballos and Medellin 1988; Reid 1997; Koopman 1994 |
| *Diclidurus ingens* | *24.7* | 70.7 | Bezerra and Cunha 2007; Ceballos and Medellin 1988; Koopman 1994 |
| *Diclidurus isabellus* | *26.7* | 54.0 | Koopman 1994 |
| *Diclidurus scutatus* | *25.8* | 56.0 | Ceballos and Medellin 1988; Sodré and Uieda 2006; Koopman 1994 |
| *Emballonura alecto* | *48.5* | 45.4 | Tate and Archbold 1939; Helgen 2002; Koopman 1994 |
| *Emballonura beccarii* | *55.8* | 39.7 | Tate and Archbold 1939; Koopman 1994 |
| *Emballonura dianae* | 32.4 | 45.6 | Hill 1985; Koopman 1994 |
| **Emballonura furax* | 41.9 | 47.5 | Tate and Archbold 1939; Hill 1985; Koopman 1994 |
| *Emballonura monticola* | 50.8 | 44.0 | Tate and Archbold 1939; Koopman 1994 |
| *Emballonura raffrayana* | *42.6* | 41.8 | Tate and Archbold 1939; Koopman 1994 |
| *Emballonura semicaudata* | *47.3* | 41.0 | Tate and Archbold 1939 |
| *Emballonura serii* | *48.4* | 47.5 | https://www.gbif.org/species/4265215 |
| **Mosia nigrescens* | *60.2* | 35.0 | Helgen 2002 |
| *Paremballonura atrata  (previously Emballonura)* | *51.2* | 38.0 | Tate and Archbold 1939; Koopman 1994 |
| *Peropteryx kappleri* | 32.0 | 48.7 | Reid 1997; Koopman 1994 |
| *Peropteryx leucoptera* | *32.2* | 44.0 | McDonough et al. 2010 |
| *Peropteryx macrotis* | 37.4 | 42.3 | Yee 2000; Koopman 1994 |
| *Peropteryx trinitatis* | *41.6* | 39.8 | Santos, Silva, and Faria 1992 |
| ***Rhynchonycteris naso*** | **89.7** | **37.8** | Plumpton and Jones 1992; Reid 1997; Koopman 1994 |
| *Saccolaimus flaviventris* | 19.0 | 75.0 | Koopman 1994 |
| **Saccolaimus mixtus* | *22.3* | 64.5 | Koopman 1994 |
| **Saccolaimus peli* | *20.8* | 89.5 | Koopman 1994 |
| *Saccolaimus saccolaimus* | 23.0 | 71.5 | Koopman 1994 |
| **Saccopteryx antioquensis* | *55.0* | 37.1 | Muñoz and Cuartas 2001 |
| *Saccopteryx bilineata* | 45.8 | 46.2 | Yancey, Goetze, and Jones 1998a; Koopman 1994; Reid 1997 |
| *Saccopteryx canescens* | *66.0* | 38.0 | Koopman 1994 |
| *Saccopteryx gymnura* | *55.7* | 34.0 | Koopman 1994 |
| *Saccopteryx leptura* | 49.5 | 39.3 | Yancey, Goetze, and Jones 1998b; Koopman 1994 |
| **Taphozous achates* | 23.2 | 63.1 | Kitchener et al. 1993 |
| *Taphozous australis* | *21.6* | 65.0 | Koopman 1994 |
| *Taphozous georgianus* | 17.0 | 68.0 | Colket and Wilson 1998; Koopman 1994 |
| **Taphozous hamiltoni* | *23.0* | *65.0* | Colket and Wilson 1998; Koopman 1994 |
| **Taphozous hildegardeae* | *25.4* | *65.0* | Colket and Wilson 1998; Koopman 1994 |
| *Taphozous hilli* | 27.5 | 67.5 | Colket and Wilson 1998; Koopman 1994 |
| **Taphozous kapalgensis* | 24.0 | 60.5 | Colket and Wilson 1998; Koopman 1994 |
| *Taphozous longimanus* | *28.2* | *58.5* | Colket and Wilson 1998; Koopman 1994 |
| **Taphozous mauritianus* | 25.3 | 61.3 | Dengis 1996; Koopman 1994 |
| *Taphozous melanopogon* | 29.1 | 64.7 | Wei et al. 2008; Koopman 1994 |
| *Taphozous nudiventris* | *23.7* | *72.5* | Colket and Wilson 1998 |
| **Taphozous perforatus* | 31.4 | 62.0 | Colket and Wilson 1998; Koopman 1994 |
| **Taphozous theobaldi* | *23.6* | *71.0* | Colket and Wilson 1998; Koopman 1994 |
| **Taphozous troughtoni* | 22.0 | 72.5 | Colket and Wilson 1998; Koopman 1994 |

Species with an asterisk (*) were not included because no phylogenetic information was present. The source of the echolocation call peak frequency is Collen 2012.

**Table S5:** Frequencies and amplitudes per syllables type needed for acoustic range calculation.

| Syllable type | number of  amplitude  measurements | peak frequency (kHz)  (loudest Harmonic) | approx.  amplitude  (dB re. 20 μPa at 1 m) | acoustic range  (m) | Predominant selection pressure |
| --- | --- | --- | --- | --- | --- |
| *Hook* | 211* | 98.33 | 100 | 13 | natural |
| *FM Hook* | 11 | 45.90 | 81 | 22 | sexual |
| *Cascade* | 8 | 25.84 | 73 | 38 | sexual |
| *Chatter* | 12 | 96.72 | 80 | 9 | natural |
| *Trill* | 12 | 45.08 | 73 | 19 | natural |
| *Trill (second harmonic)* | 12 | 90.16 | 73 | 8 | natural |
| *Isolation call* | 7 | 82.50 | 78 | 10 | natural |
| *FMT1 Syllable* | 10 | 69.09 | 77 | 12 | natural |
| *FMT2 Syllable* | 10 | 51.55 | 80 | 19 | natural |
| *Downsweep* | 10 | 37.62 | 77 | 26 | sexual |
| *LFP* | 10 | 22.44 | 72 | 44 | sexual |
| *V* | 9 | 40.72 | 73 | 21 | natural |
| *Ripple* | 8 | 32.99 | 77 | 31 | natural |
| *Ripple (second harmonic)* | 8 | 65.99 | 77 | 13 | natural |
| *Whine* | 8 | 41.15 | 88 | 30 | sexual |
| *Upsweep* | 10 | 35.71 | 81 | 31 | natural |
| *Inverted V* | 10 | 32.14 | 80 | 35 | natural |
| *Frequency jump* | 10 | 29.96 | 85 | 44 | sexual |
| *LFC* | 10 | 9.94 | 60 | 55 | natural |
| *LFS* | 10 | 15.42 | 74 | 77 | sexual |
| *Screech* | 9 | 28.29 | 65 | 26 | sexual |
| *Long composite syllable* | 7 | 35.17 | 78 | 30 | sexual |
| *Short composite syllable* | 10 | 21.72 | 73 | 48 | sexual |

Please note that the calculation is not fully reliable below 20 kHz. In an equal ratio, the first and second harmonic of the Trill and Ripple are emphasized. Thus, we calculated the acoustic range for both peak frequencies. **Hooks* were simultaneously measured with all other syllable types.

| syllable type | Predicted syllable type [%] | | | | | | | | | | | | | | | | | | | |  |
| --- | --- | --- | --- | --- | --- | --- | --- | --- | --- | --- | --- | --- | --- | --- | --- | --- | --- | --- | --- | --- | --- |
|  | 1 | 2 | 3 | 4 | 5 | 6 | 7 | 8 | 9 | 10 | 11 | 12 | 13 | 14 | 15 | 16 | 17 | 18 | 19 | total |  |
|  |  |  |  |  |  |  |  |  |  |  |  |  |  |  |  |  |  |  |  |  |  |
| 1 | 92.9 | 0 | 0 | 4 | 0 | 0 | 0 | 0 | 0 | 0 | 0 | 3.6 | 0 | 0 | 0 | 0 | 0 | 0 | 0 | 100 |  |
| 2 | 0 | 88.0 | 0 | 8 | 0 | 4 | 0 | 0 | 0 | 0 | 0 | 0 | 0 | 0 | 0 | 0 | 0 | 0 | 0 | 100 |  |
| 3 | 0 | 0 | 100.0 | 0 | 0 | 0 | 0 | 0 | 0 | 0 | 0 | 0 | 0 | 0 | 0 | 0 | 0 | 0 | 0 | 100 |  |
| 4 | 20.7 | 17.2 | 0 | 62.1 | 0 | 0 | 0 | 0 | 0 | 0 | 0 | 0 | 0 | 0 | 0 | 0 | 0 | 0 | 0 | 100 |  |
| 5 | 0 | 0 | 0 | 0 | 85.4 | 2.1 | 2.1 | 8.3 | 0 | 0 | 2.1 | 0 | 0 | 0 | 0 | 0 | 0 | 0 | 0 | 100 |  |
| 6 | 0 | 8.0 | 0 | 4 | 4 | 64.0 | 12.0 | 0 | 0 | 0 | 0 | 8.0 | 0 | 0 | 0 | 0 | 0 | 0 | 0 | 100 |  |
| 7 | 4.2 | 0 | 0 | 0 | 4 | 0 | 91.7 | 0 | 0 | 0 | 0 | 0 | 0 | 0 | 0 | 0 | 0 | 0 | 0 | 100 |  |
| 8 | 0 | 0 | 0 | 0 | 10 | 0 | 0 | 67.3 | 0 | 0 | 6.1 | 4.1 | 0 | 0 | 4 | 0 | 0 | 0 | 8.2 | 100 |  |
| 9 | 0 | 0 | 5 | 5 | 0 | 0 | 0 | 0 | 72.7 | 0 | 0 | 0 | 0 | 18 | 0 | 0 | 0 | 0 | 0 | 100 |  |
| 10 | 0 | 0 | 0 | 0 | 0 | 0 | 0 | 0 | 0 | 100.0 | 0 | 0 | 0 | 0 | 0 | 0 | 0 | 0 | 0 | 100 |  |
| 11 | 0 | 0 | 0 | 0 | 0 | 0 | 0 | 8 | 0 | 0 | 84.6 | 2 | 0 | 0 | 0 | 0 | 0 | 0 | 5.8 | 100 |  |
| 12 | 0 | 0 | 0 | 0 | 0 | 0 | 0 | 0 | 0 | 0 | 0 | 96.0 | 0 | 0 | 4 | 0 | 0 | 0 | 0 | 100 |  |
| 13 | 0 | 0 | 0 | 0 | 0 | 0 | 0 | 1.7 | 1.7 | 0 | 3.4 | 3.4 | 44.8 | 12.1 | 6.9 | 13.8 | 0 | 0 | 12.1 | 100 |  |
| 14 | 0 | 0 | 0 | 0 | 0 | 0 | 0 | 0 | 0 | 0 | 0 | 0 | 20.0 | 76.0 | 0 | 0 | 4.0 | 0 | 0 | 100 |  |
| 15 | 0 | 0 | 0 | 0 | 0 | 0 | 0 | 0 | 0 | 0 | 0 | 10.3 | 15.4 | 0 | 71.8 | 2.6 | 0 | 0 | 0 | 100 |  |
| 16 | 0 | 0 | 0 | 0 | 0 | 0 | 0 | 0 | 0 | 0 | 0 | 0 | 18.4 | 0 | 7.9 | 63.2 | 10.5 | 0 | 0 | 100 |  |
| 17 | 0 | 0 | 0 | 0 | 0 | 0 | 0 | 0 | 0 | 0 | 3.8 | 0 | 0 | 0 | 0 | 11.5 | 84.6 | 0 | 0 | 100 |  |
| 18 | 0 | 0 | 0 | 0 | 0 | 0 | 0 | 0 | 0 | 0 | 0 | 0 | 0 | 0 | 0 | 0 | 0 | 87.0 | 13.0 | 100 |  |
| 19 | 0 | 0 | 0 | 0 | 0 | 0 | 0 | 4.8 | 0 | 0 | 4.8 | 0 | 0 | 0 | 4.8 | 0 | 0 | 4.8 | 81.0 | 100 |  |

**Table S6:** DFA classification success for tonal syllable types.

The chance level for correct classification was 5.26%. Syllable types 1 = *Hook*, 2 = *FM hook*, 3 = *Cascade*, 4 = *Chatter*, 5 = *FMT2*, 6 = *Downsweep*, 7 = *FMT1*, 8 = *LFP*, 9 = *Trill*, 10 = *Echolocation call*, 11 = *V*, 12 = *Isolation call*, 13 = *Ripple*, 14 = *Whine*, 15 = *Inverted V*, 16 = *Upsweep*, 17 = *Frequency jump*, 18 = *LFC*, 19 = *LFS*

**Table S7:** Assessment of model fit for the DFA of tonal syllables.

| Assessment of model fit | DF1 | DF2 | DF3 | DF4 | DF5 |
| --- | --- | --- | --- | --- | --- |
| eigenvalue | 10.9 | 4.5 | 3.6 | 1.9 | 1.3 |
| explained variation [%] | 44.5 | 18.4 | 14.7 | 7.8 | 5.3 |
| Wilk's λ | 0.000 | 0.001 | 0.005 | 0.023 | 0.066 |
| Chi-squared (for p < 0.0001) | 5624.1 | 4156.8 | 3146.2 | 2239.8 | 1605.3 |

**Table S8:** DFA classification success for non-tonal syllable types.

| syllable type | Predicted syllable type [%] | | | |  |
| --- | --- | --- | --- | --- | --- |
|  | 1 | 2 | 3 | total |  |
|  |  |  |  |  |  |
| 1 | 87.5 | 8.3 | 4.2 | 100 |  |
| 2 | 0.0 | 91.7 | 8.3 | 100 |  |
| 3 | 0.0 | 0.0 | 100.0 | 100 |  |

The chance level for correct classification was 33.3%. 1 = *Screech*, 2 = *Short composite syllable*, 3 = *Long composite syllable*

**Table S9:** Assessment of model fit for the DFA of non-tonal syllables.

| Assessment of model fit | DF1 | DF2 |
| --- | --- | --- |
| eigenvalue | 4.6 | 3.4 |
| explained variation [%] | 57.6 | 42.4 |
| Wilk's λ | 0.040 | 0.226 |
| Chi-squared (for p < 0.0001) | 191.437 | 88.409 |

## Syllable type descriptions

Some of the syllable types can occur as monosyllabic calls. These are described in the supplement section “vocalization type description”.

*FM hook:* This syllable occurs mainly in *Songs,* occasionally in *Chatter* and *Crescendo*. This syllable belongs to the “hook-like” class and strongly resembles a *Hook*. The major difference to the *Hook* is the prolonged, straight frequency modulated (FM) down-sweep at the end of the *FM hook*. The peak frequency of the first harmonic is 42.8 ± 3.12 kHz. The bandwidth of the first harmonic can reach 25 kHz in contrast to a maximum of 12 kHz in a normal *Hook*. There are transitions to *Chatter* syllables.

*Cascade:* A cascade syllable belongs to the “hook-like” class and is similar to an *FM hook*, but it has a larger bandwidth (up to 35 kHz in the first harmonic), a bend in the FM part, a longer duration (13.7 ± 2.3 ms) and lower peak frequency (31.6 ± 3.7 kHz, 1st harmonic). It looks like an *FM hook* connected to a low-frequency pulse. Occasionally, *Cascades* transit into *FM hooks* or into a *Trill.* Two to three harmonics occur. The first or second harmonic is emphasized. *Cascades* are almost exclusively produced in *Songs*.

*Downsweep:* In contrast to the *FMT-downsweep*, the *Downsweep* does not occur in trains. A *Downsweep* is a downward modulated syllable. Sometimes a short upwards modulated part precedes the downwards modulated part (similar to *Hooks*). The *Downsweep* maximum frequency (35-45 kHz in the 1st harmonic) and the peak frequency (36.3 ± 2.4 kHz) are lower than that of a *Hoo*k. Usually, two harmonics can be detected, with the first one being emphasized. The *Downsweep* is part of many complex vocalizations, for example, the *Song, Crescendo* and *Variable complex calls*.

*Low frequency pulse (LFP):* The *Low frequency pulse* is the shortest syllable with a duration of 2.0 ± 1.0 ms. Additionally, the peak frequency is low (22.3 ± 5.2 kHz) but not the lowest of the repertoire. The frequency is often downward modulated. There are three to five harmonics with either the first or second being emphasized. Due to the short duration, the many harmonics and frequency modulation (ca. 8 kHz bandwidth), the *Low frequency pulse* sometimes appears noisy. This syllable is regularly part of *Songs*, *Crescendos* and *Variable complex calls*.

*Low frequency syllable (LFS):* This syllable has a low peak frequency of 15.1 ± 2.1 kHz. In contrast to the *Low frequency pulse*, it has a longer duration (9.1 ± 2.9 ms) and its curvature resembles more that of a *Ripple* than a *Downsweep* (lower bandwidth with ca. 6 kHz versus 13 kHz). The two to six harmonics are clearly distinguishable. The first one is emphasized. This syllable is also regularly part of *Songs*, *Crescendos* and *Variable complex calls*.

*Short composite syllable:* The most distinctive feature of a *Short composite syllable* is the combination of a noisy and a tonal part. Compared to the *Long composite syllable*, the *Short composite syllable* is four times shorter (15.0 ± 2.9 ms) and more variable. The noisy part may follow the tonal one. Sometimes the noisy part is not completely harsh, but shows subharmonics. This syllable is quite common, especially in *Variable complex calls*. It is often produced in series. The tonal part often has more than four harmonics. Its peak frequency is 27.2 ± 5.2 kHz.

*Trill*: A *Trill* is a long tonal syllable (46.6 ± 10.6 ms) that resembles an irregular sine wave, featuring multiple frequency modulations. It appears like regularly spaced *FM hooks/Hooks* connected. A *Trill* sometimes transits into a *Chatter* when not all elements are connected. A *Trill* is always preceded by a *Hook* or *FM hook*. The bandwidth can vary strongly between *Trills* (one of the highest standard deviations in bandwidth with ca. 8 kHz). *Trills* can either drop in frequency (from 50 to 20 kHz) or remain within a 30 - 50 kHz range, averaging a peak frequency of 43.7 ± 4.3 kHz. It has two harmonics, one of which is emphasized. *Trills* may occur in series, singly, or as part of complex vocalizations.

*Ripple:* The frequency of this syllable is quasi-constantly shaped with some modulations. *Ripples* vary strongly in peak frequency (31.3 ± 9.2 kHz), duration (27.1 ± 21.4 ms) and number of harmonics (2-5 harmonics). Either of the first two is emphasized. *Ripples* occur as a syllable in complex calls and often transit into *Whines, Frequency jumps, Upsweeps,* and *Inverted Vs*. Sometimes ripples are produced in series with an interval of 0.15 ± 0.05 s.

*Inverted V*: This tonal syllable type is characterized by an increase and following decrease in frequency. It is produced by both sexes and occasionally in series. *Inverted Vs* can be variable, e.g. the maximum frequency of the first harmonic ranges between 25-45 kHz, and two to five harmonics have been recorded. This call can transit into *Upsweeps* and *Downsweeps*. As a syllable, it is present in complex calls, such as *Crescendo* and *Songs*.

*Decrescendo:* Similar to a *Crescendo*, a *Decrescendo* is a sequence of syllables that change their frequency one by one. In contrast to the *Crescendo*, the *Decrescendo* is gradually decreasing in frequency and does not occur as an independent vocalization. Most commonly, it is part of a *Song*. The single syllables within the decrescendo are variable. On average, it lasts 155.74 ± 10.06 ms and has a total peak frequency of 46.02 ± 12.53 kHz.

*Dip:* The *Dip* is a combination of a *Crescendo* and a *Decrescend*o. This syllable sequence only exists in *Songs*.

## Vocalization type descriptions

### Monosyllabic vocalizations

*Frequency jump*: This rare call is relatively long (23.9 ± 10.5 ms) and has a quasi-constant or shallow upsweep frequency curvature. It features an abrupt jump to a higher frequency, usually in the middle, without any pause. *Frequency jumps* mainly occur in series, often in combination with *Ripples* and *Upsweeps*. Transitions to the latter are common. Two to four harmonics are detectable, with the first being emphasized. *Frequency jumps are* often produced in an aggressive context and simultaneously with other social calls of conspecifics. Occasionally, they appear in *Songs*.

*Screech:* The *Screech* is the only noisy (harsh) vocalization. It is always emitted in an aggressive context of two conspecifics, regardless of sex. This call is very common, possibly because there are many behaviors which lead to aggressive interaction. For example, sometimes females refuse copulation, group members do not keep the inter-individual-distance, or dominant males prevent other males from copulating. A screech is audible for humans, louder than the average vocalization of *R. naso*, and one of the longest monosyllabic calls (74.8 ± 26.4 ms). Additionally, it has a broad bandwidth (ca. 80 kHz), ranging between 5 - 90 kHz regularly, with no clear harmonics and a peak frequency of 28.1 ± 7.2 kHz. *Screeches* are often emitted in series at 171 ± 60 ms intervals and can transit to *Long composite calls*.

*Long composite call*: If the long composite syllable occurs as a monosyllabic call, it is a *Long composite call*. This call consists of two distinctive parts that merge seamlessly into one another. The first part of this call is a *Screech* (noisy). The second part is tonal, frequency modulated, and resembles a *Ripple, Trill,* or *Upsweep*. The tonal part tends to increase in frequency. The proportion between the two parts varies strongly. The long composite call shares many features with the *Screech*, for example a long duration (64.6 ± 25.9 ms), large bandwidth (ca. 65 kHz), and inter-call-interval in series (120.7 ± 60.0 ms). It is also produced exclusively during aggressive interaction and can transit into a S*creech*.

*Whine: Whines* are tonal, quasi-constant calls, which show only marginal frequency modulations (bandwidth ca. 3 kHz) and a comparatively long duration (45.4 ± 18.5 ms). This call is rarely produced and also not present as a syllable in complex calls. It has two harmonics; the first one is emphasized. It is likely uttered in an aggressive context.

### Multisyllabic simple vocalizations/trains

*Echolocation call: Echolocation calls* are produced by all sexes during flight or in situations of high awareness, for example when a conspecific enters the colony or a predator is close. This tonal call consists of a short up- and down-sweep part of nearly equal bandwidth (ca. 3 kHz). The echolocation call is the highest call, with its peak frequency around 100 kHz. On very rare occasions, a first harmonic at ca. 50 kHz can be detected. *Echolocation calls* may rarely be part of complex calls. The *Echolocation call* is more stereotypic than other vocalizations of *R. naso*.

*Hook: Hooks* are the most frequent call (and syllables) and are produced by all sexes. A *Hook* starts similarly to the *Echolocation call*, but its down-sweep is more frequency modulated and can reach 42 kHz in its first harmonic. The second harmonic is emphasized almost exclusively, with its peak frequency at 84 kHz. Sometimes the first harmonic is not detectable. They are most often emitted in succession with a constant interval of 0.05 ± 0.02 s. Typically *Hooks* are produced during rocking behaviour, before and during copulations, and when roosting in the night roosts. As a syllable, *Hooks* frequently combine with *FMT*, and occur in C*rescendos, Variable complex calls,* and *Songs*.

*Chatter:* This very common multisyllabic call is a train of three to seven syllables, which are similar to *Hooks* or *FM hooks*. They are emitted regularly with an interval of 20.9 ± 9 ms. In contrast to the repetition of multiple *Hooks*, the syllables of *Chatter* are much closer to each other and usually last a bit longer (6.5 ± 1.4 ms). Either the first or second harmonic is emphasized. Both sexes produce *Chatter*. They occur singly or in series and are mostly produced during rocking behavior and directly before flight departure. *Chatter* can be part of complex vocalizations like the *Song*. Transitions to *Trills* are common.

*Upsweep*: This common call is produced by both sexes, produced in series and is characterized by an upwards modulated frequency. In contrast to the *Downsweep*, the *Upsweep* has a longer duration (21.7 ± 8 ms) and shallower frequency modulation (bandwidth ca. 8 kHz). The peak frequency of the first harmonic is at 35.5 ± 4.1 kHz. Two to five harmonics can be detected. One of the first two harmonics is emphasized. The series of upsweeps is most often produced during the occurrence of a *Hook* train of one or multiple conspecifics. It is a variable call and transits regularly to multiple other vocalization/ syllable types, e.g. to *Frequency jumps, Ripples,* and *Inverted Vs*. As a syllable, it is also present in complex calls. No clear behavioural context could be assigned.

*FMT1*: The *FMT1* stands for a frequency modulated train 1. This common train consists of 6 to 19 equal downsweeps, which are highly frequency modulated and of very short duration (ca. 1 ms). In spectrograms, they appear as vertical pulses, with at least three harmonics, the second being emphasized. In total this call lasts 80.42 ± 21.57 ms and has an inter-syllable-interval of 8.6 ± 1 ms and a peak frequency of ca. 76 kHz. The bandwidth of the first harmonic is circa 8 kHz. Most of the time, one or multiple *Hooks* precede an *FMT1*. Sometimes it is followed by *Hooks* or *Chatter*. A *FMT1* is also often used in other complex calls like the *Song*, for example. Both sexes produce this call, but its behavioral context is unclear.

*FMT2*: This common frequency modulated train also consists of 4-6 similar, highly frequency-modulated downsweeps. However, these downsweep syllables are greater in duration (ca. 3 ms) and bandwidth (ca. 9 kHz) in comparison to the *FMT1*-downsweeps. The inter-syllable interval is 21 ± 12 ms. Transitions and mixing between the *V* and *FMT2* occur. Three to four harmonics are reproduced. The second harmonic is emphasized, and the peak frequency is ca. 55 kHz. The *FMT2* call is also often embedded in *Hooks* and no clear behavioral context is known. Similarly to the *FMT1*, it can occur in complex calls and is often preceded by another syllable as well. It is produced by both sexes.

*Low frequency call* (*LFC*): The *LFC* is a rare tonal call with a very low peak frequency of around 10 kHz, the lowest of all calls. It consists of multi-second repetitions of a long syllable (ca. 24 ms) with a quasi-constant frequency, sometimes slightly upward-modulated. Typically, two harmonics are produced, with either one emphasized. The *LFC* has the lowest amplitude. Mothers tend to rock with their three-to-four-week-old babies for multiple minutes apparently to encourage pups to let go of the teat and perch on their own. During this period, *LFCs* are emitted, likely by the mothers. It is possible that the rocking and vocalizing motivate the pup to leave the mother.

*Isolation call*: The *Isolation call* is produced by pups within minutes after birth. These calls are recorded regularly during the transition from one teat to the next. They are also produced during the night by the pups, while the mother is foraging and absent. *Isolation calls* are relatively short (ca. 5 ms) and have a short quasi-constant or upwards modulated part, which is followed by a downsweep. Usually, three harmonics can be detected, while the second is emphasized. The peak frequency of the second harmonic is at ca. 70 kHz and, thus, considerably lower in comparison to other hook-like calls (2^nd^ harmonic at ca. 100 kHz).

### Multisyllabic complex vocalizations

*Complex call with V train*: This complex call consists of a train of three to six V-shaped syllables (*V*), which is embedded in a start and end syllable. The end syllable is nearly always an *FMT2*-downsweep. There are frequent transitions and mixtures between the *V train* and *FMT2*. For example, a train could consist of 3 *Vs* and 2 *FMT2-*downsweeps. The inter-syllable-interval of the *V-Train* is identical to the interval of *FMT2* (21 ± 7 ms). The peak frequency is ca. 38 kHz. The *V-Train* and end-syllable have three to six harmonics, while the first or second harmonic is emphasized. The start syllable is more variable. In most cases, it is a *Hook*, but there are variations with *Inverted V, Downsweep,* and *Ripple* syllables. This call is produced in a mother-pup-interaction. The pup utters it most often when it is attached to the mother.

*Pup vocal sequences*: This vocalization type is highly variable. It is only produced by pups that perch on their own (age: three to four weeks) in their birth colony. *Pup vocal sequences* are usually multi-second long and include a series of *Hooks*, which continuously vary in their frequencies (like a wave). However, *Upsweeps, V, FMT2*, and *Inverted V*s are also common. A pup usually repeats a *Pup vocal sequences* multiple times, which can last several minutes in total. Only pups that frequently perch alone but are still of an age to nurse produce this call. The series is very long, its intensity increases towards the end, and it usually only ends as soon as the young have body contact with their mother. The *Pup vocal sequences* share some similarities with the *Song* (multiple-second length and up-and-down modulation with multiple syllables); however, the inter-syllable interval is longer (61.75 ± 35.21 ms), fewer syllable types are used, and it is seldom audible to humans.

*Variable complex call*: This variable complex call is a group of calls, that match in length (300-800 ms) and syllable composition (at least three syllable types), but do not have defined syllable types or order. Often, the *Variable complex call* starts with syllables of low frequencies (e.g. *LFP*), includes an *FMT* or *V,* and ends with higher syllables (e.g. *Chatter, Trill, Hook*). The mean peak frequency is at ca. 46 kHz. The syllables are often unevenly distributed and grouped. Both sexes produce these calls. We frequently observed adults producing this call after an individual enters the social group. Also, pups perching on their own can vocalize *Variable complex calls*. If they do so, they often repeat a similarly structured *Variable complex call* for multiple times with an interval of 0.5 to three seconds.

*Crescendo*: A *Crescendo* is a very common multisyllabic call of 9 to 20 syllables, in which each syllable has a higher frequency than the previous one until a plateau is reached. The length of the plateau varies strongly. The maximum frequency of the first syllable is usually lower than 30 kHz. The last syllable has a maximum frequency of ca. 50 kHz and it is usually a *Hook*. The central syllables cannot be assigned to a certain syllable type but are rather transitions between different types. They are similar to *Inverted Vs*, *Upsweeps, Hooks,* or *Downsweeps*. Two to three harmonics are produced and the first or second is emphasized. The peak frequency is ca. 62 kHz. Both sexes produce this call. A *Crescendo* is often part of a *Song*.

*Song:* This vocalization is the longest complex vocalization with a mean duration of 3 s, on average 100 syllables and 13 syllable types. It is produced only by adult males, predominantly in the mating season and at dawn (occasionally also during night). *Songs* are produced in a mating context, but not during copulation. During the vocalization, the male (and other group members) stays motionless, with only his mouth opening widely. The *Song* varies highly in its structure. The duration, syllable types, and the order of syllable types vary strongly. Many syllable types are often used in other vocalizations (*Hook, Crescendo, FMT1,* and others), but some are especially prominent in the *Song* and rare in other vocalizations: *Decrescendo, Dip, FM hook, Cascade, Downsweep, LFP.* Most of the *Song* syllables have two harmonics, but others reach up to five. The first or second one is usually emphasized. This vocalization is one of the few ones of Proboscis bats that humans can hear. It has a large bandwidth (between 8 - 50 kHz in the first harmonic) and frequently reaches a minimum frequency of less than 10 kHz. The mean peak frequency is ca. 43 kHz. In future, we will investigate the song in more detail.

**Table S10:** Results of the DFA for testing of individual information encoded in the vocalization type *Hook.*

| Assessment of model fit | DF1 | DF2 | DF3 | DF4 | DF5 |
| --- | --- | --- | --- | --- | --- |
| eigenvalue | 2.445 | 0.641 | 0.494 | 0.223 | 0.201 |
| explained variation [%] | 61.1 | 16.0 | 12.3 | 5.6 | 5.0 |
| Wilk's λ | 0.081 | 0.278 | 0.456 | 0.681 | 0.833 |
| Chi-squared (for p < 0.0001) | 205.259 | 104.454 | 64.068 | 31.329 | 14.913 |

DF = discriminant function

**Table S11:** Comparison between *Echolocation call* syllables, *Hook* syllables and average tonal syllables of *R. naso*.

| Vocalization type | | duration (s) | interval (s) | peak freq (kHz,  1. Harmonic) | min freq (kHz) | max freq (kHz) | bandwidth (kHz) |
| --- | --- | --- | --- | --- | --- | --- | --- |
| *Echolocation call* | mean ± STD | 0.007 ± 0.002 | 0.05 ± 0.02 | 49.94 ± 0.90 | 49.67 ± 0.90 | 50.72 ± 0.86 | 2.66 ± 0.34 |
|  | N | 29 | 29 | 29 | 29 | 29 | 13 |
| *Hook* | mean ± STD | 0.005 ± 0.001 | 0.05 ± 0.01 | 49.17 ± 0.84 | 48.24 ± 1.23 | 49.98 ± 0.87 | 8.79 ± 2.37 |
|  | N | 28 | 28 | 28 | 28 | 28 | 28 |
| Average tonal syllable | mean ± STD | 0.018 ± 0.021 | 0.06 ± 0.06 | 32.42 ± 12.02 | 31.53 ± 11.50 | 37.26 ± 13.10 | 10.47 ± 2.52 |
|  | N | 681 | 299 | 681 | 681 | 681 | 424 |

The largest difference in acoustic parameters between Echolocation calls and Hooks is the bandwidth

**Table S12:** Potential predators and prey of Proboscis bats and their maximum hearing frequency.

| Predator group | Predator | Maximum  hearing freq. (kHz) | Source |
| --- | --- | --- | --- |
| Birds | Barn owls  (*Tyto alba pratincola*) | 13 | Dyson, Klump, and Gauger 1998 |
|  | Red-tailed hawks  (*Buteo jamaicensis)* | 8 | McGee et al. 2019 |
|  | Bald eagle  (*Haliaeetus leucocephalus*) | 5.7* | McGee et al. 2019 |
|  | Tucan (Ramphastidae) | *not included* | *unknown* |
| Mammal*** | Squirrel monkey  (*Saimiri sciureus*) | 11 | Wienicke, Häusler, and Jürgens 2001 |
|  | Spidermonkey  (*Ateles paniscus*) | 16 | Coleman 2009 |
|  | Howler monkey  (*Alouatta palliataa*) | 22 | (Ramsier et al. 2019) |
|  | Capuchine monkey  (*Sapajus apella*) | 26 | Ramsier, Vinyard, and Dominy 2017 |
|  | Racoon (*Procyon lotor*) | 40 | Wollack 1965 |
|  | Domestic cat (*Felis catus*) | 85 | Heffner and Heffner 1985 |
| Bats | Spectral bat  (*Vampyrum spectrum*) | 95 | *estimated* |
|  | Big-eared woolly bat  (*Chrotopterus auritus*) | 117** | Yoh et al. 2020 |
|  | Fringe-lipped bat  *(Trachops cirrhosus)* | 90** | Surlykke et al. 2013; Yoh et al. 2020 |
|  | Greater spear-nosed bat  (*Phyllostomus hastatus*) | 105 | Koay et al. 2002 |
|  | White-throated round-eared bat (*Lophostoma silvicola****)*** | 85** | Yoh et al. 2020 |
| Others | Unspecific snake species | 1 | Young 1997 |
|  | Bullfrog  (*Rana catesbeiana*) | 3 | Werner et al. 2009 |
|  | Orb-weaving spiders  (*Larinioides sclopetarius*) | 1 | Zhou et al. 2022 |
|  | Giant centipedes | Vibration only | *unknown* |
|  | Unspecific fish species | 3 | Popper and Hawkins 2021 |

* = assuming similar hearing range for eagles from central and South America, ** = derived from call parameter, *** It is known that non-domesticated felids (e.g. ocelot) also prey on Emballonurid bats in Central and South America (Tinoco and Camacho 2015). Some species of Felidae can hear up to 65 kHz (Kitchener et al. 2010).

References

Arroyo-Cabrales, Joaquín, and J. Knox Jones. 1988. ‘*Balantiopteryx Io* and *Balantiopteryx Infusca*’. *Mammalian Species*, no. 313: 1–3.

Arroyo-Cabrales, Joaquin, and J. Knox Jones. 1988. ‘*Balantiopteryx Plicata*’. *Mammalian Species*, no. 301 (January): 1. https://doi.org/10.2307/3504133.

Bernard, Enrico. 2003. ‘*Cormura Brevirostris*’. *Mammalian Species* 737 (December): 1–3. https://doi.org/10.1644/737.

Bezerra, Alexandra M. R., and Adriano S. Cunha. 2007. ‘First Occurrence of the Genus Diclidurus Wied, 1820 (Emballonuridae: Emballonurinae) in Central Brazil’. *Chiroptera Neotropical* 13 (1).

Ceballos, Gerado, and Rodrigo A. Medellin. 1988. ‘*Diclidurus Albus*’. *The American Society of Mammalogists*, Mammalian Species, vol. 316 (June): 1–4.

Coleman, Mark N. 2009. ‘What Do Primates Hear? A Meta-Analysis of All Known Nonhuman Primate Behavioral Audiograms’. *International Journal of Primatology* 30 (1): 55–91. https://doi.org/10.1007/s10764-008-9330-1.

Colket, Elizabeth, and Don E. Wilson. 1998. ‘*Taphozous Hildegardeae*’. *Mammalian Species*, no. 597: 1–3.

Collen, Alanna. 2012. ‘The Evolution of Echolocation in Bats: A Comparative Approach’. University College London.

Dengis, Carol A. 1996. ‘*Taphozous Mauritianus*’. *Mammalian Species*, no. 522: 1–5.

Dunlop, Jenna. 1997. ‘*Coleura Afra*’. *Mammalian Species*, no. 566: 1–4.

Dyson, M. L., G. M. Klump, and B. Gauger. 1998. ‘Absolute Hearing Thresholds and Critical Masking Ratios in the European Barn Owl: A Comparison with Other Owls’. *Journal of Comparative Physiology A: Sensory, Neural, and Behavioral Physiology* 182 (5): 695–702. https://doi.org/10.1007/s003590050214.

Helgen, KM. 2002. ‘Notes on a Collection of Mammals from the Moluccas, with New Geographic Records’. *Mammalian Biology* 67 (1): 51–54.

Hice, Christine L., and Sergio Solari. 2002. ‘First Record of *Centronycteris Maximiliani* (Fischer, 1829) and Two Additional Records of *C. Centralis* Thomas, 1912 From Peru’. *Acta Chiropterologica* 4 (2): 217–20. https://doi.org/10.3161/001.004.0209.

Hill, J. E. 1985. ‘Records of Bats (Chiroptera) from New Guinea, with the Description of a New Hipposideros (Hipposideridae)’. *Mammalia* 49 (4). https://doi.org/10.1515/mamm.1985.49.4.525.

Kitchener, Andrew C., Blaire Van Valkenburgh, and Nobuyuki Yamaguchi. 2010. ‘Felid Form and Function’. In *Biology and Conservation of Wild Felids*. CHAPTER 3.

Kitchener, DJ, LH Schmitt, S. Hisheh, RA How, and NK Cooper. 1993. ‘Morphological and Genetic Variation in the Bearded Tomb Bats (Taphozous: Emballonuridae) of Nusa Tenggara, Indonesia’. *Mammalia* 57 (1): 63–83.

Koay, Gimseong, Karen S. Bitter, Henry E. Heffner, and Rickye S. Heffner. 2002. ‘Hearing in American Leaf-Nosed Bats. I: *Phyllostomus Hastatus*’. *Hearing Research* 171 (1–2): 96–102. https://doi.org/10.1016/S0378-5955(02)00458-6.

Koopman. 1994. *Chiroptera: Systematics. Handbook of Zoology.* Vol. 8. Part 60: Mammalia. Walter de Gruyter.

McDonough, Molly M., Burton K. Lim, Adam W. Ferguson, Carson M. Brown, Santiago F. Burneo, and Loren K. Ammerman. 2010. *Mammalia, Chiroptera, Emballonuridae, Peropteryx Leucoptera Peters, 1867 and Peropteryx Pallidoptera Lim, Engstrom, Reid, Simmons, Voss and Fleck, 2010: Distributional Range Extensions in Ecuador*.

McGee, JoAnn, Peggy B. Nelson, Julia B. Ponder, Jeffrey Marr, Patrick Redig, and Edward J. Walsh. 2019. ‘Auditory Performance in Bald Eagles and Red-Tailed Hawks: A Comparative Study of Hearing in Diurnal Raptors’. *Journal of Comparative Physiology A* 205 (6): 793–811. https://doi.org/10.1007/s00359-019-01367-9.

Muñoz, Javier, and Carlos A. Cuartas. 2001. ‘*Saccopteryx Antioquensis n. Sp.*  (Chiroptera: Emballonuridae) Del Noroeste de Colombia’. *Actualidades Biológicas* 23 (75): 53–61.

Plumpton, David L., and J. Knox Jones. 1992. ‘*Rhynchonycteris Naso*’. *Mammalian Species*, no. 413 (December): 1. https://doi.org/10.2307/3504230.

Popper, Arthur N., and Anthony D. Hawkins. 2021. ‘Fish Hearing and How It Is Best Determined’. *ICES Journal of Marine Science* 78 (7): 2325–36. https://doi.org/10.1093/icesjms/fsab115.

Ramsier, Marissa A., Andrew J. Cunningham, May R. Patiño, Kenneth E. Glander, and Nathaniel J. Dominy. 2019. ‘Audiograms of Howling Monkeys: Are Extreme Loud Calls the Result of Runaway Selection?’ Preprint. https://doi.org/10.1101/539023.

Ramsier, Marissa A., Christopher J. Vinyard, and Nathaniel J. Dominy. 2017. ‘Auditory Sensitivity of the Tufted Capuchin (*Sapajus Apella*), a Test of Allometric Predictions’. *The Journal of the Acoustical Society of America* 141 (6): 4822–31. https://doi.org/10.1121/1.4986940.

Reid, Fiona. 1997. *A Field Guide to the Mammals of Central America and Southeast Mexico*. Oxford University Press.

Rickye S. Heffner and Henry E. Heffner. 1985. ‘Hearing Range of the Domestic Cat’. *Hearing Research* 19: 85–88.

Santos, Adriano Silva dos, Daiana Cardoso Silva, and Karina De Cassia Faria. 1992. ‘*Peropteryx Trinitatis* Miller, 1899 (Chiroptera, Emballonuridae): First Record in Central Brazil and Revised Distribution Map’. *Check List* 12 (6). https://doi.org/10.15560/12.6.1992.

Simmons, Nancy B., and Charles O. Handley. 1998. ‘A Revision of *Centronycteris* Gray (Chiroptera: Emballonuridae) with Notes on Natural History’. *AMERICAN MUSEUM NOVITATES*, no. 3239 (August): 28.

Sodré, Miriam M., and Wilson Uieda. 2006. ‘First Record of the Ghost Bat *Diclidurus Scutatus* Peters (Mammalia, Chiroptera, Emballonuridae) in São Paulo City, Brazil’. *Revista Brasileira de Zoologia* 23 (3): 897–98. https://doi.org/10.1590/S0101-81752006000300042.

Starrett, Andrew. 1972. ‘*Cyttarops Alecto*’. *Mammalian Species*, no. 13: 1–2.

Surlykke, Annemarie, Lasse Jakobsen, Elisabeth K. V. Kalko, and Rachel A. Page. 2013. ‘Echolocation Intensity and Directionality of Perching and Flying Fringe-Lipped Bats, *Trachops Cirrhosus* (Phyllostomidae)’. *Frontiers in Physiology* 4. https://doi.org/10.3389/fphys.2013.00143.

Tate, G. H. H., and Richard Archbold. 1939. ‘Results of the Archbold Expeditions. NO. 23, A Revision of the Genus Emballonura (Chiroptera)’. *THE AMERICAN MUSEUM OF NATURAL HISTORY New York City*, no. 1035 (July): 14.

Thomas, Oldfield. 1915. ‘LI.— *Notes on Bats of the Genus* Coleura’. *Annals and Magazine of Natural History* 15 (90): 576–79. https://doi.org/10.1080/00222931508693673.

Tinoco, Nicolás, and María Alejandra Camacho. 2015. ‘Registro de Murciélagos Depredados Por Leopardus Pardalis (Carnivora: Felidae) En El Oriente Ecuatoriano’. *Revista Biodiversidad Neotropical* 5 (2): 105. https://doi.org/10.18636/bioneotropical.v5i2.230.

Wei, Li, Naijian Han, Libiao Zhang, et al. 2008. ‘Wing Morphology, Echolocation Calls, Diet and Emergence Time of Black-Bearded Tomb Bats (*Taphozous Melanopogon*, Emballonuridae) from Southwest China’. *Acta Chiropterologica* 10 (1): 51–59.

Werner, Y. L., J. Pylka, H. Schneider, M. Seifan, W. Walkowiak, and U. Werner-Reiss. 2009. ‘Function of the Sexually Dimorphic Ear of the American Bullfrog, *Rana Catesbeiana*: Brief Review and New Insight’. *Journal of Experimental Biology* 212 (14): 2204–14. https://doi.org/10.1242/jeb.027516.

Wienicke, Häusler, and Jürgens. 2001. ‘Auditory Frequency Discrimination in the Squirrel Monkey’. *Journal of Comparative Physiology A: Sensory, Neural, and Behavioral Physiology* 187 (3): 189–95. https://doi.org/10.1007/s003590100189.

Wollack, C. Harold. 1965. ‘Auditory Thresholds in the Raccoon (*Procyon Lotor*).’ *Journal of Auditory Research* (US) 5 (2): 139–44.

Woodman, Neal. 2003. ‘New Record of the Rare Emballonurid Bat New Record of the Rare *Centronycteris Centralis* Thomas, 1912 in Costa Rica, with Notes on Feeding Habits’. *Caribbean Journal of Science* 39 (3): 399–402.

Yancey, Franklin D., Jim R. Goetze, and Clyde Jones. 1998a. ‘*Saccopteryx Bilineata*’. *Mammalian Species*, no. 581 (June): 1. https://doi.org/10.2307/3504459.

Yancey, Franklin D., Jim R. Goetze, and Clyde Jones. 1998b. ‘*Saccopteryx Leptura*’. *Mammalian Species*, no. 582 (June): 1. https://doi.org/10.2307/3504379.

Yee, Donald A. 2000. ‘*Peropteryx Macrotis*’. *Mammalian Species* 2000 (643): 1–4.

Yoh, Natalie, Peter Syme, Ricardo Rocha, Christoph F. J. Meyer, and Adrià López-Baucells. 2020. ‘Echolocation of Central Amazonian “Whispering” Phyllostomid Bats: Call Design and Interspecific Variation’. *Mammal Research* 65 (3): 583–97. https://doi.org/10.1007/s13364-020-00503-0.

Young, Bruce A. 1997. ‘A Review of Sound Production and Hearing in Snakes, with a Discussion of Intraspecific Acoustic Communication in Snakes’. *Penn State University Press* 71 (1): 39–46.

Zhou, Jian, Junpeng Lai, Gil Menda, et al. 2022. ‘Outsourced Hearing in an Orb-Weaving Spider That Uses Its Web as an Auditory Sensor’. *Proceedings of the National Academy of Sciences* 119 (14): e2122789119. https://doi.org/10.1073/pnas.2122789119.
